# Amino acid transporters within the solute carrier superfamily: Underappreciated proteins and novel opportunities for cancer therapy

**DOI:** 10.1016/j.molmet.2024.101952

**Published:** 2024-05-03

**Authors:** Kiavash Hushmandi, Behzad Einollahi, Seyed Hassan Saadat, E. Hui Clarissa Lee, Marzieh Ramezani Farani, Elena Okina, Yun Suk Huh, Noushin Nabavi, Shokooh Salimimoghadam, Alan Prem Kumar

**Affiliations:** 1 Nephrology and Urology Research Center, Clinical Sciences Institute, Baqiyatallah University of Medical Sciences, Tehran, Iran; 2 Department of Pharmacology, Yong Loo Lin School of Medicine, National University of Singapore, Singapore, Singapore; 3 NUS Center for Cancer Research (N2CR), Yong Loo Lin School of Medicine, National University of Singapore, Singapore, Singapore; 4 NanoBio High-Tech Materials Research Center, Department of Biological Sciences and Bioengineering, Inha University, Incheon 22212, Republic of Korea; 5 Department of Urologic Sciences and Vancouver Prostate Centre, University of British Columbia, V6H3Z6, Vancouver, BC, Canada; 6 Department of Biochemistry and Molecular Biology, Faculty of Veterinary Medicine, Shahid Chamran University of Ahvaz, Ahvaz, Iran

**Keywords:** SLC, Amino acid, Cancer, Nutrient sensing, Cancer metabolism

## Abstract

**Background:**

Solute carrier (SLC) transporters, a diverse family of membrane proteins, are instrumental in orchestrating the intake and efflux of nutrients including amino acids, vitamins, ions, nutrients, etc, across cell membranes. This dynamic process is critical for sustaining the metabolic demands of cancer cells, promoting their survival, proliferation, and adaptation to the tumor microenvironment (TME). Amino acids are fundamental building blocks of cells and play essential roles in protein synthesis, nutrient sensing, and oncogenic signaling pathways. As key transporters of amino acids, SLCs have emerged as crucial players in maintaining cellular amino acid homeostasis, and their dysregulation is implicated in various cancer types. Thus, understanding the intricate connections between amino acids, SLCs, and cancer is pivotal for unraveling novel therapeutic targets and strategies.

**Scope of Review:**

In this review, we delve into the significant impact of amino acid carriers of the SLCs family on the growth and progression of cancer and explore the current state of knowledge in this field, shedding light on the molecular mechanisms that underlie these relationships and highlighting potential avenues for future research and clinical interventions.

**Major Conclusions:**

Amino acids transportation by SLCs plays a critical role in tumor progression. However, some studies revealed the tumor suppressor function of SLCs. Although several studies evaluated the function of SLC7A11 and SLC1A5, the role of some SLC proteins in cancer is not studied well. To exert their functions, SLCs mediate metabolic rewiring, regulate the maintenance of redox balance, affect main oncogenic pathways, regulate amino acids bioavailability within the TME, and alter the sensitivity of cancer cells to therapeutics. However, different therapeutic methods that prevent the function of SLCs were able to inhibit tumor progression. This comprehensive review provides insights into a rapidly evolving area of cancer biology by focusing on amino acids and their transporters within the SLC superfamily.

## Graphical abstract

**Figure:**
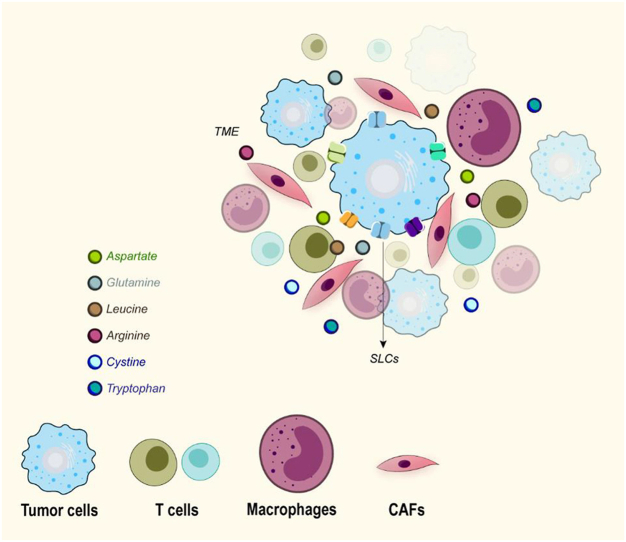

## 1 Introduction

Almost 10% (∼2000 genes) of the human genome are responsible for encoding proteins involved in membrane transportation of molecules. Gatekeepers are transporters in organelles and all types of cells, which control the import or efflux of a diversity of molecules including amino acids, nucleotides, ions, and drugs. Transporters mainly include water channels, ion channels, pump proteins, ABC transporters, and membrane-bound solute carrier (SLC) proteins. SLCs are the biggest group of transporters and more than 450 SLC proteins organized into more than 65 canonical and several non-canonical families are recognized by now. SLCs are secondary active transporters that facilitate passive transport, use ion (mainly Na^+^, Cl^−^, K^+^, or H^+^) gradients to ease uphill transports, and work as exchangers. In addition, SLCs could be found on the cell surface and organelles (mitochondria, vesicles, and melanosomes), translocating soluble molecules across cellular membranes [[1], [2], [3], [4], [5], [6], [7]]. Dysregulation of SLCs is associated with various diseases including cancer and metabolic disorders [8]. Transporters within the SLCs family which transport amino acids (SLC1, SLC6, and SLC7), sugars (SLC5 and SLC2), vitamins (SLC19), ions (SLC8, SLC22), and trace elements (SLC11, and SLC41) are involved in cancer biology [9].

Members are assigned into a family if having more than 20% amino acids sequence identity to other members of a family. Regarding the structural properties of SLCs such as having multiple domains, studying these proteins by protein purification and solubilization is challenging, hindering downstream structural investigations. It bears noting that most of the current knowledge about SLC structure and mechanism is derived through studying prokaryotic homologs [5]. The description of SLCs' structure is crucial for the rational design of activators, inhibitors, and substrates that target SLCs. Interestingly, as many of the SLCs transport charged molecules, studying the electrogenicity of SLCs is also of importance. Additionally, the redundancy of SLCs which results from their multiple isoforms, differing in their C- or N-termini, that affect protein–protein interactions, transport efficiency, transport stoichiometry, or localization should be brought into account. More importantly, redundancy in SLCs' substrates also exists, resulting in transporting several substrates by one SLC protein. However, some SLCs act specifically to transfer a certain molecule [10].

Importantly, SLCs are often mutated in various pathologies such as neurological and metabolic disorders, and cancer. Also, some marketed drugs such as diuretics, neuropsychiatric, and antidiabetic drugs are developed that target SLC transporters [8]. For instance, polymorphism in *SLC22A4/5* genes is associated with inflammatory bowel disease. Also, genetic alteration of *SLC30A8* was found to happen in diabetes. Moreover, SLC14A1 and SLC4A7 polymorphisms are accompanied by bladder cancer and abnormal blood pressure, respectively [11].

Human amino acids are categorized into different groups such as essential (Isoleucine, Leucine, Lysine, Methionine, Phenylalanine, Threonine, Tryptophan, Valine, and Histidine), non-essential (Alanine, Asparagine, Aspartate, Glutamate, and Serine), and conditionally essential (Arginine, Cysteine, Glutamine, Glycine, Proline, and Tyrosine) amino acids [12]. Amino acids are vital for the survival of cells that experience metabolism reprogramming in cancer. Also, amino acid derivatives support or inhibit tumor progression. For instance, Arginine-derived polyamines promote cancer cell proliferation, while tryptophan-derived kynurenine mediates immunosuppression. In addition, amino acids are involved in epigenetic, post-transcriptional, and protein regulation and redox balance. In addition, amino acids have a primary role in the biosynthesis of nitrogenous metabolites by providing carbon and nitrogen. Importantly, amino acids could drive mechanisms to increase ATP production by cancer cells [[13], [14], [15], [16], [17]]. Also, the proliferative drive of cancer cells is dependent on an abundant supply of amino acids. It bears noting that several cancers are auxotrophic and need even non-essential amino acids for their progression [18].

Regarding the importance of SLCs in transporting amino acids, an in-depth study of these transporters is of importance for developing next-generation SLC drugs. The current review discussed the function of SLCs which transport amino acids in cancer progression and looked at the SLC regulators.

## 2 Solute carrier family transporters

**Figure 1:**
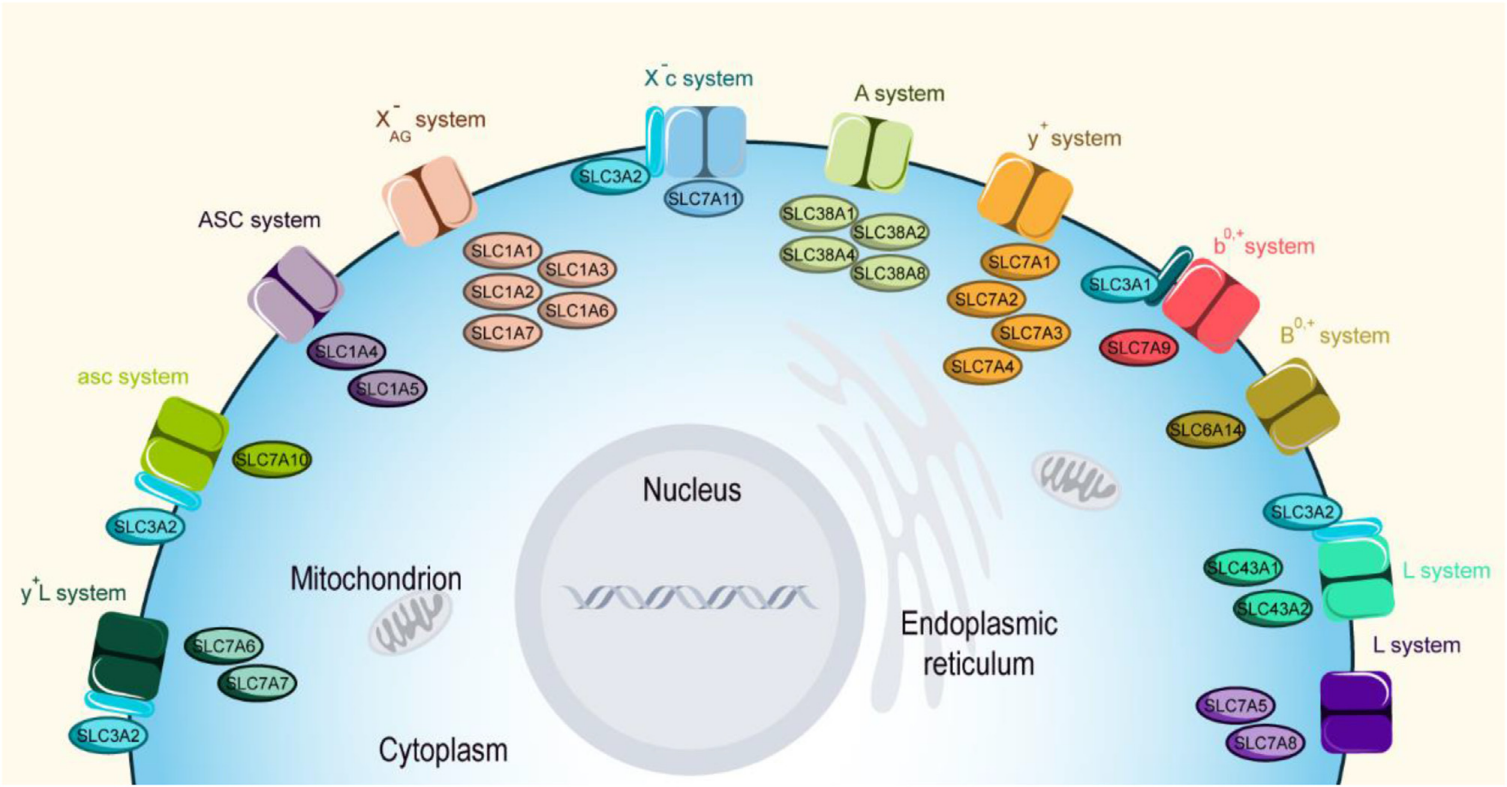
**SLCs and the systems by which they transport amino acids.** These SLCs transport amino acids in Na^+^-dependent or independent ways. Some of these transporters are heterodimerized with SLC3A1 or SLC3A2.

SLC proteins are a group of membrane cells' and organelles’ transporters which include more than 60 families, controlling the uptake and efflux of various molecules and compounds (refer to bioparadigms.org) [19]. SLCs could transport neutral, basic, or acidic amino acids. Also, they transport amino acids in a Na^+^-dependent or independent way [12]. Importantly, various transport systems have been defined for SLCs. X^−^_c_ system is a Na^+^ independent heterodimeric cystine/glutamate antiporter, composed of SLC7A11 and SLC3A2. As cystine is the precursor of cysteine, an amino acid involved in defense against oxidative damage, impairment of the X^−^_c_ system leads to cell death. Importantly, suppression of this system by compounds such as erastin, sulfasalazine, and sorafenib increases ferroptosis-mediated cell death [20]. X^−^_AG_ is a Na^+^-dependent system of the SLC1 family responsible for transporting amino acids [9]. SLC38A1, SLC38A2, SLC38A4, SLC38A8, and SLC38A10 transport system is A, dependent to Na^+^, and are responsible for transporting small, polar amino acids [9,21]. Alanine serine cysteine (ASC) system is Na^+^ dependent small neutral amino acids system of SLC1A4 and SLC1A5 which are responsible for exchanging essential (threonine), non-essential (alanine, serine, and cysteine), and conditionally essential (glutamine) amino acids [12,22,23]. In contrast, the asc system works independently of Na^+^, and small neutral amino acids are transported by SLC7A10/SLC3A2 (BAT1/rBAT). In addition, some of the neutral and cationic amino acids are exchanged by system b^0,+^ and SLC7A9/SLC3A1 uses this system and works independently of Na^+^. However, B^0,+^ is a Na^+^ dependent system of SLC6A14 and passages neutral amino acids. y^+^ L transporters work dependent to Na^+^ and composed of SLC7A6 and SLC3A2 (y^+^ LAT-2/4F2hc), or SLC7A7 and SLC3A2 (y^+^ LAT-1/4F2hc) which transport cationic amino acids. Similarly to X^−^_c_, asc, and y^+^ L, SLC3A2 is an accessory protein for SLC7A5 and SLC7A8 in the L system, which is a Na^+^-independent system and transports large hydrophobic neutral amino acids. Indeed, SLC7A5-11 are members of system L and are characterized as glycoprotein-associated amino acid transporters and are heterodimerized with SLC3A1 (rBAT) or SLC3A2 (4F2hc) [9,21,24]. Moreover, SLCs could be symporters (e.g. SLC6A14), antiporters (e.g. SLC7A5-8), or uniporters (e.g. SLC43A1/2) [25] (Figure 1). Most of the SLCs are broadly studied for their role in various types of cancer. However, the function of some of the SLCs in cancer is not explored yet. The next section explored the current findings on the role of amino acid transporter SLCs in cancer.

## 3 SLCs in cancer

### 3.1 SLC1A3

SLC1A3 role in gastric cancer progression has been revealed to be applied by targeting the PI3K/AKT signaling pathway [26]. PI3K belongs to a family of serine/threonine kinases that trigger the AKT. Numerous molecules such as hormones, cytokines, growth factors, non-coding RNAs, and oncogenic factors could activate PI3K/AKT signaling. This pathway is also a direct target of therapeutic methods. PI3K/AKT signaling affects all the cancer hallmarks such as apoptosis, metabolic reprogramming, metastasis, and therapy resistance to increase tumor progression [[27], [28], [29]]. In a study, it was found that aspartate availability is involved in different responses of cancer cells to inhibition of the mitochondrial electron transport chain (ETC). The authors demonstrated that cancer cell lines resistant to ETC inhibition maintain their aspartate level high using the SLC1A3 transporter, and modulation of SLC1A3 notably changed the sensitivity of cancer cells to ETC suppression [30]. Similarly, SLC1A3 activation through p53 during glutamine deprivation results in increased aspartate metabolism, followed by enhanced ETC and tricarboxylic acid (TCA) cycle activity [31]. SLC1A3 is also a downstream target for key proteins involved in cancer progression during hypoxia. For instance, hypoxia-inducible factor (HIF)-1 and HIF-2 proteins increase *SLC1A1/3* gene expression in hepatocellular carcinoma (HCC) and clear cell renal carcinoma cells (ccRCC) under hypoxic conditions to increase proliferation [31]. In solid tumors, SLC1A3 mediates resistance to l-asparaginase by fueling aspartate, glutamate, and glutamine metabolisms. In addition, its suppression was found to be associated with cancer cell cycle arrest and apoptosis, and *in vivo* experiments revealed that SLC1A3 upregulation enhanced metastasis and tumor growth [32].

### 3.2 SLC1A4

**Figure 2:**
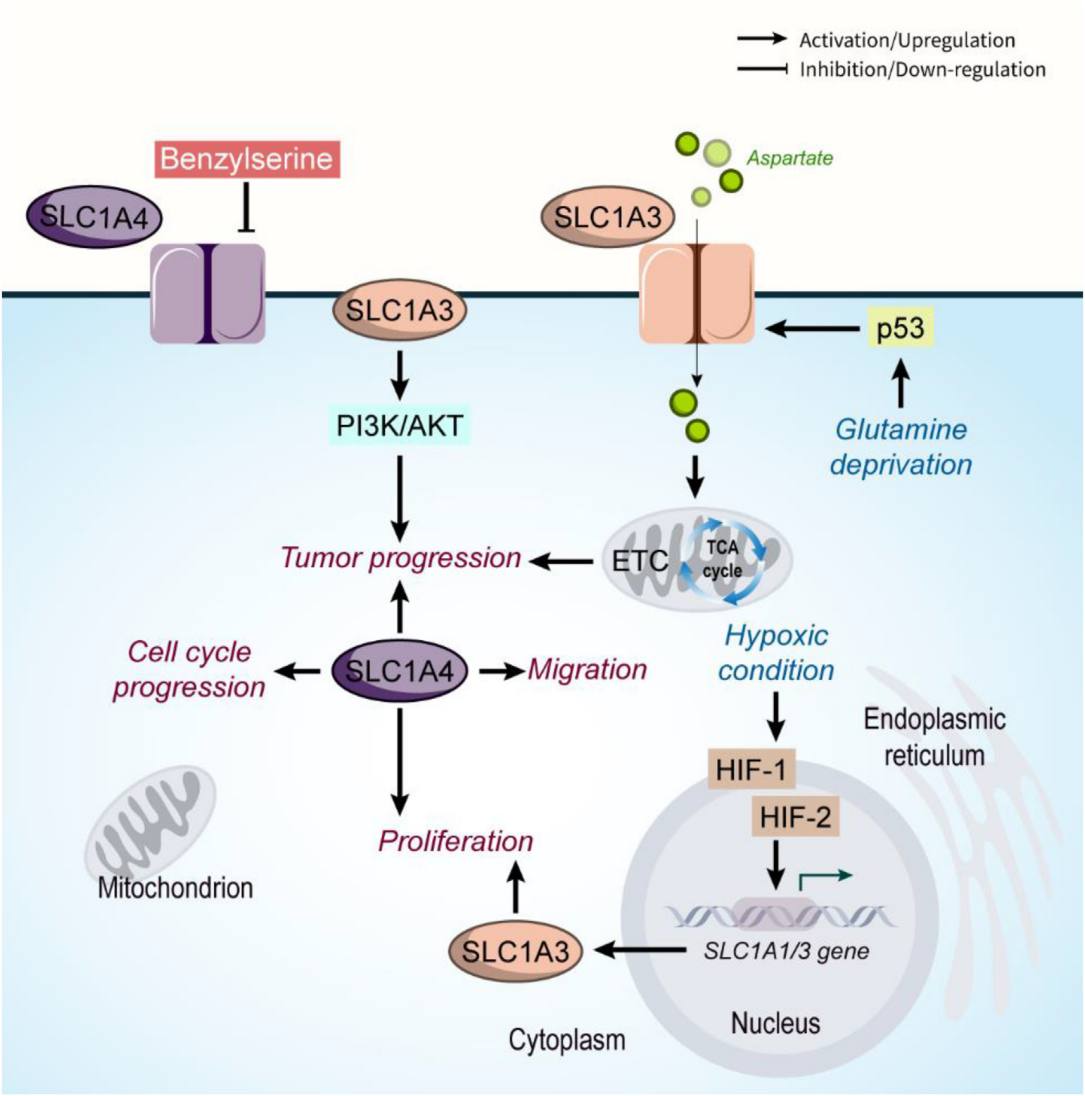
**SLC1A3 and SLC1A4 role in cancer.** SLC1A4 and SLC1A3 are transporters with tumor-promoting functions and enhance tumor cell proliferation and metastasis. Abbreviations: ETC electron transport chain, HIF hypoxia-inducible factor.

SLC1A4 is a Na^+^-dependent neutral amino acid transporter and its mRNA and protein levels increased in HCC. It was found that SLC1A4 overexpression is a powerful prognostic biomarker and is accompanied by cancer cell cycle progression, metabolism, oncogenic pathways, proliferation, and migration, while it mediates apoptosis suppression. Also, immune infiltration and immune-related chemokine expression were enhanced after SLC1A4 overexpression [33]. In pancreatic ductal adenocarcinoma (PDAC), the expression of SLC38A2 increases to promote uptake of alanine. Tumor growth of cells that lack SLC38A2 transporters was shown to be notably decreased. Interestingly, pancreatic satellite cells use transporters including SLC1A4 to increase the environmental level of alanine. This alanine cross-talk between PDAC and pancreatic satellite cells is a unique therapeutic target that affects the metabolic demands of cancer cells [34]. In another study on breast cancer cells, *SLC1A4* was detected as a gene whose expression indicated the occurrence of ferroptosis [35]. It was also found that SLC1A4 promotes rapid proliferation, invasion, and metastasis by regulating metabolic reprogramming, and also increases cancer stem cell abilities. Importantly, cells were shown high sensitivity toward anti-cancer therapies when the expression level of SLC1A4 is low [36]. Also, SLC1A4 and other transporters such as SLC7A8, SLC38A1, and SLC38A2 were detected as putative targets of an anti-cancer drug named Benzylserine in breast cancer, which their uptake activity is directly inhibited by this drug [37]. In contrast, it showed that SLC1A4 expression is downregulated in non-small cell lung cancer cells (NSCLC), while SLC38A1, SLC1A5, SLC7A5, and SLC7A11 were upregulated. However, the expression level of SLC1A4 showed a significant increase following the SLC1A5 depletion (Figure 2) [38].

### 3.3 SLC1A5

**Figure 3:**
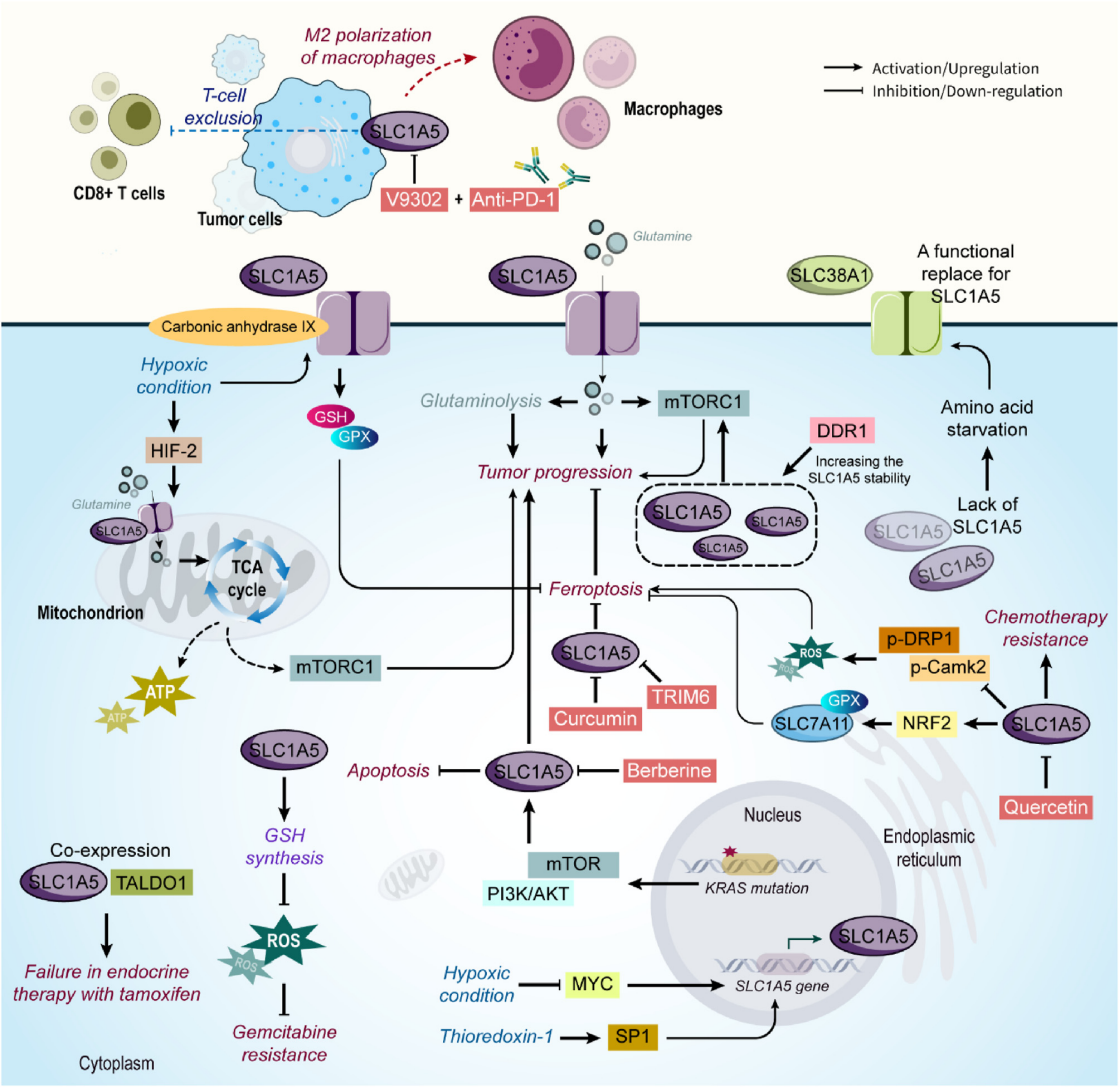
**SLC1A5 role in cancer.** SLC1A5 regulates the TCA cycle and ROS metabolism in cancer cells, enhances drug resistance, and prevents apoptosis and ferroptosis in augmenting tumor growth. Moreover, SLC1A5 affects the M2 polarization of macrophages, a function that could be suppressed by a combination of V9302 and anti-PD-1 therapy. Berberine, curcumin, and quercetin are among the natural products that effectively attenuate the function of SLC1A5 in cancer. Abbreviations: DDR1 Discoid protein domain receptor 1, GSH glutathione, mTORC1 mammalian target of rapamycin complex 1, TALDO1 Transaldolase 1, TCA tricarboxylic acid cycle.

SLC1A5, also known as alanine, serine, cysteine-preferring transporter 2 (ASCT2), mediates glutamine uptake, an essential amino acid for cancer cells. As a therapeutic method, starving cancer cells of glutamine by SLC1A5 inhibitors leads to impairment of tumor progression [39,40]. Glutamine is critical for the glutaminolysis process and activation of the mammalian target of the rapamycin complex 1 (mTORC1) pathway which is involved in cancer cell growth. In triple-negative basal-like breast cancer, SLC1A5 inhibition prevents cell proliferation and triggers rapid cell death [41]. A study revealed cytoplasmic overexpression of SLC1A5 in lung cancer is associated with cell metabolism, growth, and survival through controlling l-glutamine transportation [42]. Targeting SLC1A5 also affects autophagy and apoptosis in NSCLC, and is a putative prognostic biomarker in glutamine-dependent NSCLC [43]. Interestingly, a study revealed a variant of SLC1A5 which has an N-terminal targeting signal to localize on mitochondrial membrane, transporting glutamine. During hypoxia, HIF-2α increases the expression of the SLC1A5 variant. On the mitochondria membrane, SLC1A5 transfers glutamine into the mitochondria, mediatingthe activity of the TCA cycle followed by ATP production and increased mTORC1 activity. Also, through promoting glutathione (GSH) synthesis and suppression of ROS production, it mediates resistance to gemcitabine [44]. In colorectal cancer (CRC), KRAS mutation was associated with SLC1A5 higher activity resulting from PI3K/AKT and mTOR activity. SLC1A5 then increases cancer cell growth, invasion, and metastasis, while decreasing apoptosis [45]. Also, it was elucidated that SLC1A5 is overexpressed in HeLa epithelial cervical cancer cells and 143B osteosarcoma cells. However, the lack of SLC1A5 is not accompanied by tumor cells' growth inhibition but leads to amino acid starvation and SLC38A1 upregulation as a functional replacement for SLC1A5 [46]. Although inhibiting SLC1A5 in cancer is therapeutically useful, SLC1A5 is expressed in normal tissues which makes SLC1A5 targeting in cancer therapy very challenging. In human head and neck squamous cell carcinoma (HNSCC), an approved epidermal growth factor receptor (EGFR) antibody named cetuximab is used for treating the metastatic form of cancer. Interestingly, cetuximab decreases SLC1A5 in an EGFR expression-dependent manner through cetuximab-mediated EGFR endocytosis. As a result, intracellular uptake of glutamine and subsequent GSH synthesis is decreased. These metabolic changes increase HNSCC sensitivity to ROS-induced apoptosis. In addition, in this study, it was demonstrated that suppression of EGFR by gefitinib failed to mediate HNSCC cells' sensitivity to apoptosis induced by ROS [47]. Also, regarding the critical function of SLC1A5 in glutamine metabolism, SLC1A5 is essential for tumorigenesis in HNSCC, and it was shown that SLC1A5 prevents apoptosis and autophagy, while increasing growth and proliferation. In addition, suppression of SLC1A5 leads to inactivation of the mTORC1 pathway, increased oxidative stress, and an improved response of HNSCC to cetuximab [48]. Similarly, in metastatic CRC, inhibition of SLC1A5 results in an improved response to cetuximab by increasing the proteasomal degradation of EGFR and decreasing the expression of nuclear EGFR [49]. A synthetic glucose analog named 2-deoxyglucose suppresses glycolysis, which leads to thioredoxin-1 overexpression in CRC. In turn, thioredoxin-1 prevents the cytotoxicity of 2- deoxyglucose. However, inhibition of thioredoxin-1 by PX-12 is in favor of 2-deoxyglucose anti-cancer function. Furthermore, thioredoxin-1 binds to SP1 to increase the promoter activity of SLC1A5, thereby upregulation of SLC1A5. Overall, the study suggested a novel adaptive mechanism of glycolytic suppression induced by 2-deoxyglucose, in which GSH is increased through thioredoxin-1/SP1/SLC1A5 axis [50]. In ccRCC, the high expression level of SLC1A5 was related to poor overall survival and prognosis [22]. The prognostic role of SLC1A5 also was assessed in stomach adenocarcinoma and its association with tumor-infiltrating immune cells and immune checkpoints in the tumor microenvironment (TME) was revealed [51]. In HCC, a transmembrane receptor tyrosine kinase named Discoid protein domain receptor 1 (DDR1) interacts with SLC1A5, regulates its stability, and thereby affects its downstream target, mTORC1 in increasing HCC progression [52]. Overexpression of SLC1A5 was also demonstrated in glioblastoma. SLC1A5 expression was revealed to be associated with immune response, while silencing SLC1A5 negatively affects proliferation and invasion, and decreases the infiltration and M2 polarization of tumor-associated macrophages. Moreover, suppression of SLC1A5 by V9302 promotes the efficacy of anti-PD-1, making SLC1A5 a valuable prognostic biomarker and therapeutic target for glioblastoma [53]. Interestingly, inspiratory hyperoxia is shown to prevent metastasis through the downregulation of SLC1A5. During hyperoxia (60% oxygen), MYC expression is decreased which leads to the downregulation of its downstream target gene, *SLC1A5*. Then, the disturbance in glutamine uptake and catabolism, and inhibition of invasion and metastasis is happened [54]. Also, the results of a study by Alfarsi et al. revealed the importance of considering and targeting SLC1A5 in cancer therapy. They show that SLC1A5 co-expression with Transaldolase 1 (TALDO1) metabolic enzyme in receptor-positive breast cancer cells is associated with failure in endocrine therapy with tamoxifen [55]. SLC1A5 could also be a target for phytochemicals. For instance, berberine suppresses liver cancer cell growth by inhibiting the SLC1A5 transporter [56]. Also, curcumin exerts anticancer activity against breast cancer cells by promoting SLC1A5-mediated ferroptosis [57]. Inhibition of SLC1A5 by quercetin in CRC promotes sensitivity of cancer cells to chemotherapy [58]. Furthermore, quercetin binds to the SER-343, SER-345, ILE-423, and THR-460 residues of SLC1A5 to suppress its expression and induce ferroptosis in gastric cancer cells. By inhibiting SLC1A5, quercetin prevents the nuclear translocation of NRF2 and inhibits xCT/GPX4. Moreover, SLC1A5 suppression by quercetin leads to the activation of p-Camk2 and p-DRP1 and increased ROS levels. Quercetin/SLC1A5 axis also increases the iron content in cancer cells [59]. In hypoxic tumor cells, it was demonstrated that Carbonic anhydrase IX interacts with SLC1A5 to maintain redox homeostasis through the GSH/GPX4 axis. Inhibition of Carbonic anhydrase IX function leads to high lipid peroxidation and ferroptosis in cancer cells [60]. In lung cancer, Tripartite motif 6 (TRIM6) interacts with SLC1A5 to increase its degradation, thereby promoting ferroptosis and cancer cell death [61]. In SCC, SLC1A5 high expression was revealed to be associated with CD8^+^ T-cell exclusion in the TME [62] (Figure 3).

### 3.4 SLC3A2

**Figure 4:**
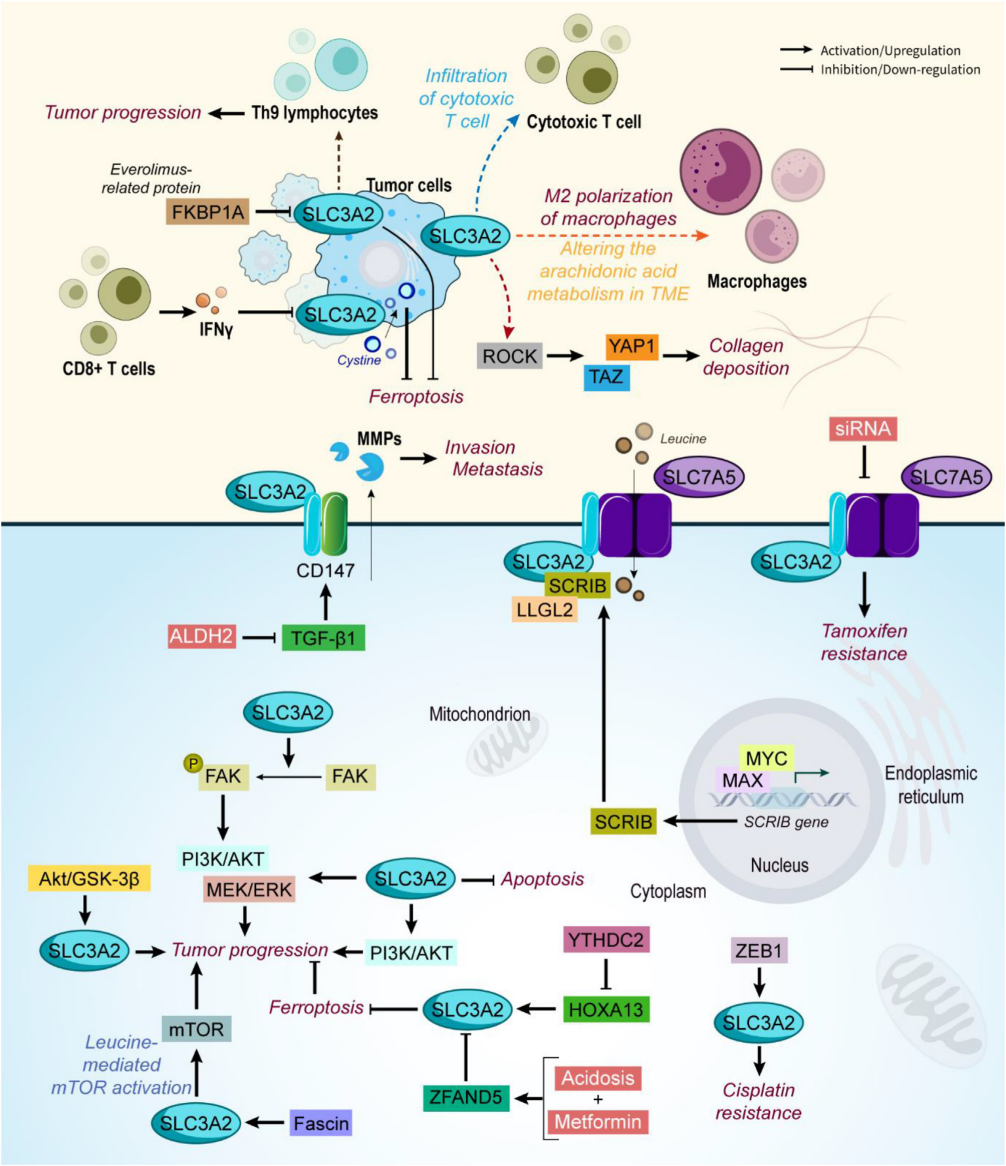
**SLC3A2 role in cancer.** SLC3A2 is heterodimerized with some of the other SLCs, thus affecting several oncogenic pathways and molecules, including PI3K/AKT, mTOR, and MEK/ERK. SLC3A2 is involved in the regulation of ferroptosis, apoptosis, invasion, metastasis, and drug resistance. In the TME, SLC3A2 also increases the M2 polarization of macrophages and prevents the anti-cancer function of T cells. Abbreviations: FKBP1A FK506-binding protein 1A, HOXA13 Homeo box A13, IFNγ interferon-gamma, MMP metalloproteinase, ROCK Rho kinase, SCRIB Scribble, TAZ transcriptional coactivator with PDZ-binding motif, YAP1 Yes-associated protein 1, ZEB1 Zinc finger E-box-binding homeobox 1.

In breast cancer, SLC3A2 expression was found to be associated with poor patient outcomes [63]. ccRCC cells' migration, proliferation, and survival were also demonstrated to be associated with CD98hc (SLC3A2) overexpression through its integrin binding domain. Thereby, FAK phosphorylation happened, and signaling pathways including PI3k/Akt and MEK/ERK are activated in increasing ccRCC progression [64]. Similarly, SLC3A2 promotes tumor growth in osteosarcoma through the PI3K/AKT signaling pathway [65]. In oral squamous cell carcinoma (OSCC), upregulation of SLC3A2 was found to be related to advanced stages and poor survival in patients. A high level of SLC3A2 increases cancer cell proliferation, invasion, and migration, while its knocking down increases apoptosis in cancer cells [66]. Also, in estrogen-receptor (ER) positive breast cancer cells, it was revealed that co-expression of SLC7A5/SLC3A2 leads to increased proliferation in cancer cells and is associated with poor prognosis, while their knocking down using siRNA-sensitized breast cancer cells to tamoxifen. Overall, considering the expression of SLC7A5/SLC3A2 in breast cancer patients could predict failure in endocrine therapy and guide the choice of other alternative therapies [67]. Induced by estrogen signaling in a MYC-dependent manner, cell polarity proteins Scribble (SCRIB) has tumor-promoting function and mediates tumor cell proliferation and endocrine resistance to tamoxifen in ER-positive breast cancer cells. In these cells, *SCRIB* gene expression is increased through estrogen-induced MYC-MAX binding to its promoter/enhancer. For exerting its function, SCRIB interacts with SLC3A2 and forms a quaternary complex with LLGL2-SLC7A5 to regulate leucine uptake and promote the proliferation of ER-positive breast cancer cells [68]. Also, SLC3A2 overexpression in lung cancer was related to tumor-associated macrophages (TAMs) and poor prognosis. It was found that silencing SLC3A2 in lung cancer cells inhibits M2 polarization of macrophages, changes the cancer cell's metabolism, and alters metabolites such as arachidonic acid in the TME. Overall, SLC3A2 induces macrophage phenotypic reprogramming through arachidonic acid by acting as a metabolic switch [69]. Interestingly, Zinc finger E-box-binding homeobox 1 (ZEB1) mediates abnormal expression of SLC3A2 in inducing chemoresistance to cisplatin in ovarian cancer cells [70]. The SLC3A2-NRG1 fusion protein is composed of a SLC3A2 (SLC3A2 heavy chain) transmembrane domain and the EGF-like domain of the neuregulin 1(NRG1) protein (NRG1 III-β3 form) which its overexpression has been seen in cancer progression. In a study, it was demonstrated that the SLC3A2-NRG1 fusion gene is involved in lung cancer cell proliferation and tumor growth which is mediated through the generation of ERBB2–ERBB3 heterocomplex and activation of the PI3K/ERK/mTOR signaling pathway. Thus, in SLC3A2-NRG1–positive tumors, targeting ERBB2 and ERBB3 could be a promising target [71]. Consistent with previous studies, similar results were also reported in a study of invasive mucinous adenocarcinoma of the lung (IMA) [72]. Also, regarding the importance of SLC3A2-NRG1 fusion in lung cancer, in another study, it was shown that KRAS enhances tumor growth in SLC3A2-NRG1–positive lung cancer cells through ADAM17-mediated NRG1 cleavage. Following the shedding of NRG1, Ras/Raf/MEK/ERK and ERBB/PI3K/Akt/mTOR pathways are promoted [73]. In a study, it was found that SLC3A2 is expressed in a broad range of human cancers but its expression in normal tissues is restricted to the kidney, testis, and cerebellum, making this protein a tumor-associated antigen. Pellizzari and coworkers generate IgE monoclonal antibody and chimeric antigen receptor (CAR) T cell immunotherapies each targeting SLC3A2. It was demonstrated that SF-25 IgE prevents tumor growth without inducing type I hypersensitivity by triggering basophil activation in the blood of cancer patients *ex vivo*, supporting the safe therapeutic administration of the antibody. Also, SLC3A2-specific CAR T cells were shown to promote interferon-γ and interleukin-2 synthesis *in vitro* and enhance overall survival, while decreasing tumor growth. More importantly, the study confirmed no weight loss and no signs of cytokine release syndrome [74]. Also, Chen et al. stated that SLC3A2 is an immuno-oncogenic factor that its expression is accompanied by infiltration of cytotoxic T cells, but not other immune cells among breast cancer TME. During the processes of everolimus-inducing ferroptosis in breast cancer cells, an everolimus-related protein named FK506-binding protein 1A (FKBP1A) negatively regulates the SLC3A2 expression to promote anti-proliferation effect of Th9 lymphocytes [75]. In CRC cells, SLC3A2 shows overexpression and regulatory functions such as cell proliferation, migration, and EMT. Moreover, it was found that Akt/GSK-3β pathway regulates migration inhibitory factor (MIF) and SLC3A2 expression *in vivo* and *in vitro* [76]. Alcohol consumption is one of the causes of liver cancer. Aldehyde dehydrogenase 2 (ALDH2) has a key role in alcohol metabolism, and inhibits hepatocarcinogenesis, while its deficiency is associated with a high risk of HCC. Basigin (BSG; CD147) is a transmembrane glycoprotein with high expression levels in liver cancer cells which promotes proliferation and invasion through increasing the secretion of matrix metalloproteinase (MMP). Interestingly, SLC3A2 and CD147 interact to form the CD147-CD98hc complex, regulating cell metabolism, proliferation, and invasion. In liver cancer, SLC3A2 promotes the ceramide/sphingosine/sphingosine 1-phosphate axis in increasing liver cancer progression. To inhibit liver cancer progression, ALDH2 inhibits BSG via the TGF-β1 pathway, indirectly suppressing SLC3A2 expression [77]. The effect of SLC3A2 in TME was also studied and it was found that genetic deficiency of SLC3A2 prevents Ras-driven skin carcinogenesis. Beyond its intrinsic effect on cancer cell proliferation, SLC3A2 increases the stiffness of the TME and augments the capacity of cells to respond to matrix rigidity. For these purposes, SLC3A2 increases Rho kinase (ROCK) activity to promote matrix stiffness and nuclear relay of mechanical signals via YAP/TAZ signaling. It was found that silencing SLC3A2 decreases ROCK activity, YAP/TAZ signaling, and mCTGF, confirming the role of SLC3A2 in collagen deposition. SLC3A2 promotes both intra- and extra-cellular processes to enhance tumor progression. More importantly, this study also revealed that deletion of SLC3A2 after tumor induction leads to tumor regression [78]. In human lung squamous cell carcinoma (LUSC) and lung adenocarcinoma (LUAD), SLC3A2 not only induces tumorigenesis through the MEK/ERK signaling pathway but also could be used as a prognostic biomarker [79]. Also, in LUAD, Homeo box A13 (HOXA13) which is a transcriptional factor augments SLC3A2 expression in increasing cancer progression. Interestingly, HOXA13 is negatively regulated by m^6^A reader YT521-B homology containing 2 (YTHDC2). Thus, YTHDC2 indirectly inhibits SLC3A2 to induce ferroptosis in cancer cells, resulting in impairing tumor growth and inducing lipid peroxidation [80]. SLC3A2 is also upregulated by Fascin, a transcriptional factor with nuclear functions, affecting cell growth and survival through modulation of leucine-mediated mTOR activation [81]. In another study, it was shown that interferon-gamma (IFNγ)-released from CD8^+^ T cells mediates downregulation of SLC3A2. Thereby, cysteine uptake is impaired and lipid peroxidation and ferroptosis are promoted consequently. The results of this study suggest a potential therapeutic approach in which T cells promote ferroptosis in cancer cells as an anti-tumor mechanism [82]. In breast cancer, the stability of the SLC3A2 protein is altered by acidosis which results in ferroptosis in cancer cells. In addition, a combination of acidosis and metformin work synergistically to inhibit tumor progression and M1 polarization of macrophages through the ZFAND5/SLC3A2/ferroptosis pathway [83] (Figure 4).

### 3.5 SLC7A1

The oncogenic roles of SLC7A1 or cationic amino acid transporter-1 (CAT-1) have been confirmed by various studies. Mediating the bidirectional transport of cationic amino acids, SLC7A1 plays a critical role in regulating metabolic functions, such as protein synthesis, synthesis of nitric oxide (NO), polyamine biosynthesis, and interorgan amino acid flow [84]. In epithelial ovarian cancer, SLC7A1 regulates metabolism reprogramming in promoting tumor progression [85]. In hepatoblastoma, it was found that tumor suppressor speckle-type BTB/POZ (SPOP) is involved in tumorigenesis. As one of the SPOP substrates, SLC7A1 affects cell phenotype by regulating arginine metabolism, thereby regulating hepatoblastoma tumorigenesis [86]. Also, SLC7A1 high expression was confirmed in arginine-starved HCC cells. This study revealed that arginine uptake in HCC cells relies on SLC7A1, and SLC7A1 silencing slows HCC cell growth [87]. Like the previous study, SLC7A1 is involved in L-(2,3,4,5-H^3^)-arginine uptake, and its silencing of SLC7A1 was proved to result in apoptosis induction in breast cancer cells. Also, together with prolactin (PRL) and 17β-estradiol (E2), SLC7A1 induces nitric oxide synthase to produce NO, thereby increasing breast cancer cell survival [88]. In addition to breast cancer cells, increased levels of l-arginine in CRC cells by SLC7A1 also resulted in tumor cell growth. Based on this study, SLC7A1 was expressed in 70.5% of CRC samples, and silencing SLC7A1 using siRNA inhibits tumor cell growth by 20–50% [89]. Interestingly, a study revealed that SLC7A1 is the only arginine importer expressed in chronic lymphocytic leukemia cells. Furthermore, lentiviral-mediated suppression of SLC7A1 was associated with decreased cell proliferation, cell viability, and tumor growth [90]. In NSCLC, KRAS activation was found to decrease the expression level of argininosuccinate synthase 1 (ASS1), an enzyme responsible for the production of arginine from aspartate and citrulline. Thus, aspartate is turned to pyrimidine instead of arginine and is consumed for DNA replication. Inhibition of ASS1 in KRAS-mutant cells was found to impair the production of arginine, thereby rendering a dependency on SLC7A1 to uptake arginine [91]. Another study also revealed that nuclear factor erythroid 2-related factor 3 (NFE2L3, also known as NRF3) increases the entrance of arginine into lysosomes by increasing RAB5-mediated micropinocytosis, SLC7A1-mediated transport, and SLC38A9/RagC/mTORC1 pathways. After that, mitochondrial function is enhanced which leads to increased ATP production, apoptosis inhibition, and tumor cell growth [92].

### 3.6 SLC7A2

**Figure 5:**
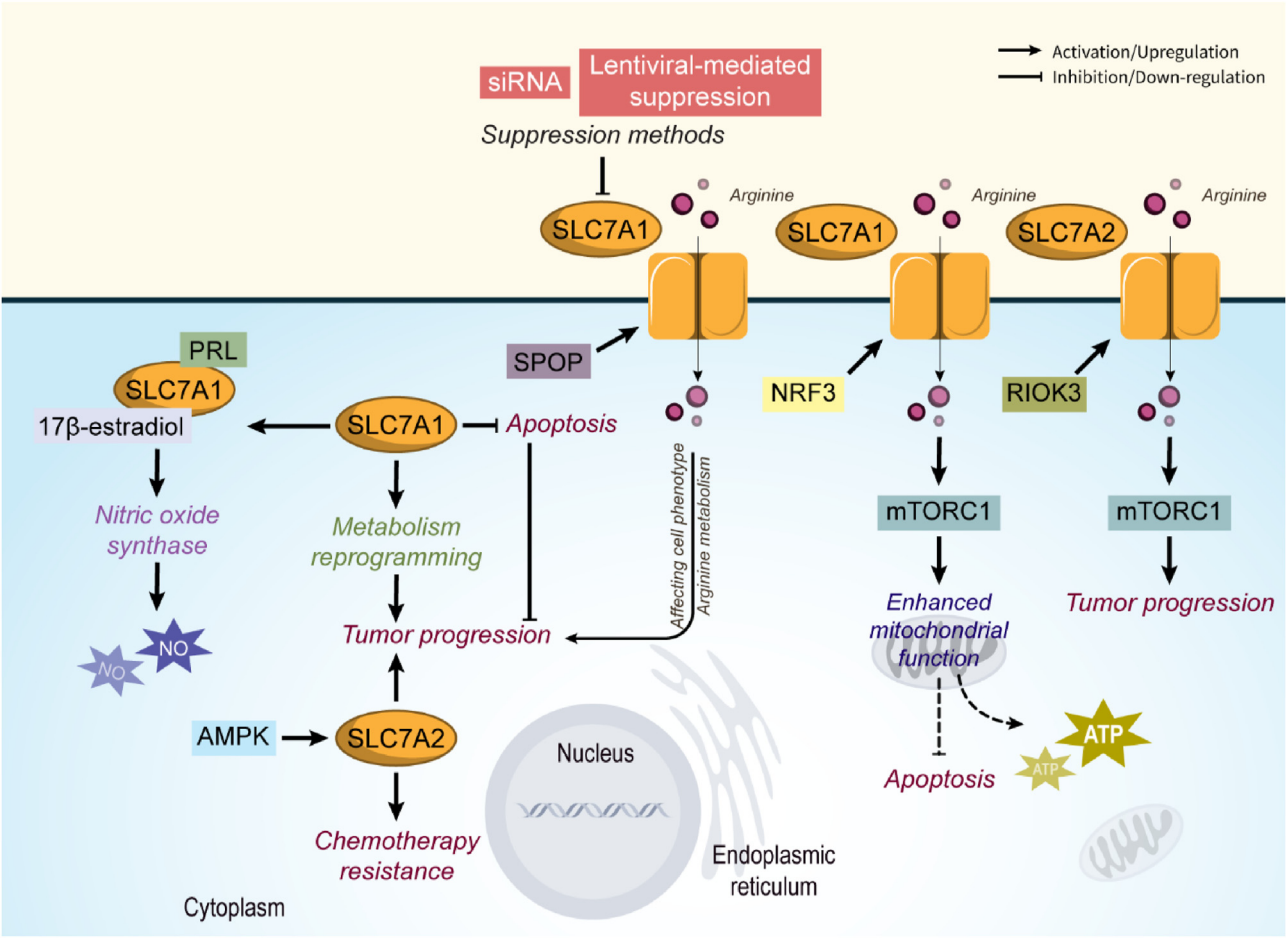
**SLC7A1 and SLC7A2 role in cancer.** SLC7A1 functions as a tumor promoter transporter by inhibiting apoptosis, regulating metabolism reprogramming, and increasing drug resistance. Abbreviations: mTORC1 mammalian target of rapamycin complex 1, RIOK3 Rio Kinase 3, SPOP speckle-type BTB/POZ.

Like SLC7A1, SLC7A2 is also a cationic amino acid transporter that its function in the regulation of cancer hallmarks is revealed in studies. But in contrast to SLC7A1, SLC7A2 expression was found to decrease in various cancer types. For instance, Jiang et al. have shown that SLC7A2 is downregulated in NSCLC. Moreover, SLC7A2 knocking down leads to NSCLC cell proliferation, and resistance to paclitaxel, cisplatin, and gemcitabine. In addition, it was demonstrated that AMPK augments SLC7A2 expression to increase the sensitivity of cancer cells to anti-cancer drugs. More importantly, SLC7A2 modulates immune infiltration and correlates to the infiltrated neutrophils, macrophages, dendritic cells, and their markers such as CD86, HLA-DPA1, and ITGAM [93]. The protecting role of SLC7A2 from colitis-associated carcinogenesis in the setting of chronic colitis was proved in a study. In SLC7A2-negative mice, tumors exhibited high levels of proinflammatory cytokines/chemokines including IL-1β, IL-3, CXCL1, CXCL5, CXCL2, CCL3, and CCL4, while the levels of IL-4, CXCL9, and CXCL10 were decreased. Also, a shift toward pro-tumorigenic M2 macrophage activation was shown in these mice [94]. On the contrary, upregulation of SLC7A2 by Rio Kinase 3 (RIOK3) was associated with increased invasion and metastasis in PDAC. For this purpose, RIOK3 promotes SLC7A2 expression to increase arginine import and mTORC1 activation [95]. Also, a study revealed the interaction of polymorphisms in the SLC7A2 gene and calcium, magnesium, or calcium: magnesium uptake ratio in CRC progression [96] (Figure 5).

### 3.7 SLC7A5

SLC7A5 or L-type amino acid transporter 1 (LAT1) is a transporter of large neutral amino acids with confirmed tumorigenic functions. LAT-1 interacts with the 4F2 cell surface antigen heavy chain (4F2hc, SLC3A2) to form a heterodimeric amino acid transporter protein complex. By regulating transcriptional and metabolic reprogramming, SLC7A5 maintains intracellular amino acid levels following KRAS activation in CRC to enhance the proliferation of KRAS-mutant cells [97]. In addition, upregulation of SLC7A5 in breast cancer is associated with decreased overall survival and enhanced proliferation through targeting AKT/mTORC1 pathway [98]. SLC7A5 also regulates cancer hallmarks in gastric cancer. It was found that CRKL depletion impaired gastric cancer progression via SLC7A5 downregulation. In contrast, ectopic expression of SLC7A5 is in favor of gastric cancer metastasis and invasion [99]. In NSCLC, it was demonstrated that nicotine increases the expression level of the SLC7A5 gene through EGFR/SERPINB5/TRIM29/NF-κB/c-Myb pathway to increase tumor progression [100]. In PDAC, a study showed that the Aly/REF export factor (ALYREF) increases the stability of JunD to promote the transcription of SLC7A5. Overexpression of SLC7A5 then resulted in increasing the import of amino acids, decreasing the level of amino acids in TME, decreasing the uptake of amino acids by CD8^+^ T cells, and then restricting their functions [101]. In lung cancer, IGF2BP2 increases the SLC7A5 mRNA stability and translation. In turn, by importing amino acids, SLC7A5 increases the expression level of IGF2BP2 in a positive feedback loop. The function of SLC7A5 also activates the AKT/mTOR pathway which then mediates the radioresistance ability of cancer cells. Importantly, it was demonstrated that GSK3β and FBW7 increase phosphorylation, ubiquitination, and proteasomal-mediated degradation of IGF2BP2, thereby inhibiting its tumor-promoting functions [102].

Also, overexpression of SLC7A5 in HER2-negative breast cancer types was shown in a study [103]. Importantly, a study revealed the relation between SLC7A5 overexpression and tamoxifen resistance in breast cancer cells, and silencing SLC7A5 expression was sufficient to increase the sensitivity of cancer cells to tamoxifen. In addition, LLGL2 regulates SLC7A5 by forming a trimeric complex with SLC7A5 and YKT6, enhancing leucine import, and thereby controlling ER+ breast cancer cell proliferation [104]. Interestingly, SLC7A5 expression is estrogen-regulated in cells sensitive to endocrine therapies, while this regulation is missing in resistant cells. It was also found that inhibition of SLC7A5 impaired the growth of endocrine-resistant breast cancer cells. Moreover, although autophagy was activated after SLC7A5 inhibition, p62 degradation failed and drug flux was inhibited [105]. The above-mentioned studies confirmed the critical role of SLC7A5 in ER+ breast cancer cells and offer a novel target for therapeutic intervention against ER+ breast cancers.

In CRC, proto-oncogene MYC regulates metabolic reprogramming to enhance cancer progression. For this purpose, MYC increases the expression of tryptophan transporters SLC1A5 and SLC7A5 and the enzyme arylformamidase (AFMID), which are involved in tryptophan conversion into kynurenine. Importantly, the sensitivity of colon cancer cells to tryptophan depletion was reported to be more than normal human colonic epithelial cells, and inhibiting the enzymes involved in the kynurenine pathway leads to colon cancer cell death [106]. Also, metformin inhibits tryptophan import into CRC cells by decreasing MYC and SLC7A5, thereby restoring the availability of tryptophan for CD8^+^ T cells and increasing their cytotoxicity [107].

In an effort, compounds based on dithiazole and dithiazine were used to target SLC7A5 in the proteoliposome experimental model. In this study, it was found that these compounds interact with SLC7A5 C407 residue to inhibit its function, and this inhibition was impaired in SLC7A5 mutant C407. Also, treatment of SiHa cells with these compounds resulted in cell death [108]. Importantly, a study was revealed that residues F252, S342, C335 plays critical role in substrate recognition, while C407 plays a minor role [109]. Thus, further studies may reveal the best SLC7A5 residue for targeting in cancer therapy.

Furthermore, SLC7A5 participates in forming immunosuppressing TME and inhibits the effect of immunotherapies in LUAD. Also, SLC7A5 silencing diminishes proliferation and migration in tumor. However, overexpression of SLC7A5 was demonstrated to be associated with cell cycle progression, DNA damage repair, high response to reactive oxygen species (ROS), angiogenesis, and EMT [110]. In HCC patients, high expression levels of SLC7A5 and SLC38A1 were shown to be associated with shorter survival. Yes-associated protein 1 (YAP1) and transcriptional coactivator with PDZ-binding motif (TAZ), downstream targets of the Hippo signaling pathway, regulate amino acid metabolism via upregulation of SLC7A5 and SLC38A1. Then, increased import of amino acids leads to activation of mTORC1 and tumor progression [111]. YAP and TAZ are transcriptional factors that are highly expressed in cancer, and vital for cancer initiation and growth. They enhance cancer cell proliferation and survival and promote metastasis and drug resistance, making them critical regulators in cancer [112]. Importantly, therapies lead upstream regulators to affect the Hippo pathway to activate YAP/TAZ [113].

In B16-F10 melanoma mouse models, using nanvuranlat (JPH203) or knocking down LAT-1 produced the same results including decreased cell proliferation, invasion, and metastasis to lung, spleen, and lymph nodes. Mechanistically, the expression of integrin αvβ3 which contributes to the metastasis process was shown to decrease following treatment with LAT-1 inhibitor and downregulation of the mTOR signaling pathway [114]. In thyroid cancer, pharmacological inhibition of SLC7A5 by JPH203 suppresses cancer cell proliferation and induces thyroid tumor growth arrest [115]. In pancreatic ductal adenocarcinoma cells [116] and medulloblastoma [117], LAT1 inhibition by JPH203 was also studied.

Furthermore, it was found that x-irradiation increases the import of amino acids by LAT-1. However, JPH203 prevents the radiation-induced amino acids uptake, increases the sensitivity of cancer cells to radiation, decreases mTOR activity, and promotes cells' senescence after irradiation [118]. Similarly, the administration of JPH203 leads to the inhibition of invasion and migration in renal cell carcinoma (RCC) [119]. Bestatin is an aminopeptidase inhibitor that blocks the amino acid recycling process and exerts anti-tumor activity. In a study, a competitive inhibitor of LAT-1 named 2-aminobicyclo[2.2.1]heptane-2-carboxylic acid (BCH) was demonstrated to promote the anti-proliferative effect of bestatin in ovarian cancer cell lines [120]. Also, in some studies, LAT-1 inhibitors are designed to suppress amino acid uptake by cancer cells. For instance, a novel selective LAT1 inhibitor was synthesized by Huttunen et al. which prevents the uptake of L-leucin, the LAT-1 substrate. After administration of this inhibitor, cell growth was reported to be decreased and the anti-cancer potential of cisplatin and bestatin was increased. Importantly, this inhibitor was found to be metabolically stable. In addition, the inhibitor was able to be detached from the cell surface, making this inhibition reversible [121]. Treating cancer cells with KMH-233, a LAT-1 inhibitor, was suggested to be a promising factor in cancer therapy. KMH-233 is hemocompatible and decreases the protein level of mTOR and NF-κB, while increasing apoptosis in LAT-1-expressing cancer cells. In addition, the level of l-leucine, l-Tyrosine, or L-Tryptophan amino acids in the brains of mice was not affected, which makes this inhibitor as a safe and effective anti-cancer agent [122]. Furthermore, triiodothyronine (T3) is a LAT-1 blocker, and in a study, a novel series of LAT-1 suppressors named SKN101–105 was designed based on the T3 structure which consisted of the core structure of 2-amino-3[3,5-dichloro-4-(naphthalene-1-methoxy)-phenyl]-propanoic acid and different modifications on the naphthalene. SKN103 with a modified phenyl group at the C-7 position of naphthalene suppressed LAT-1 transport function efficiently. In addition, mTOR activity and cancer cell growth were suppressed after SKN103 administration [123].

**Figure 6:**
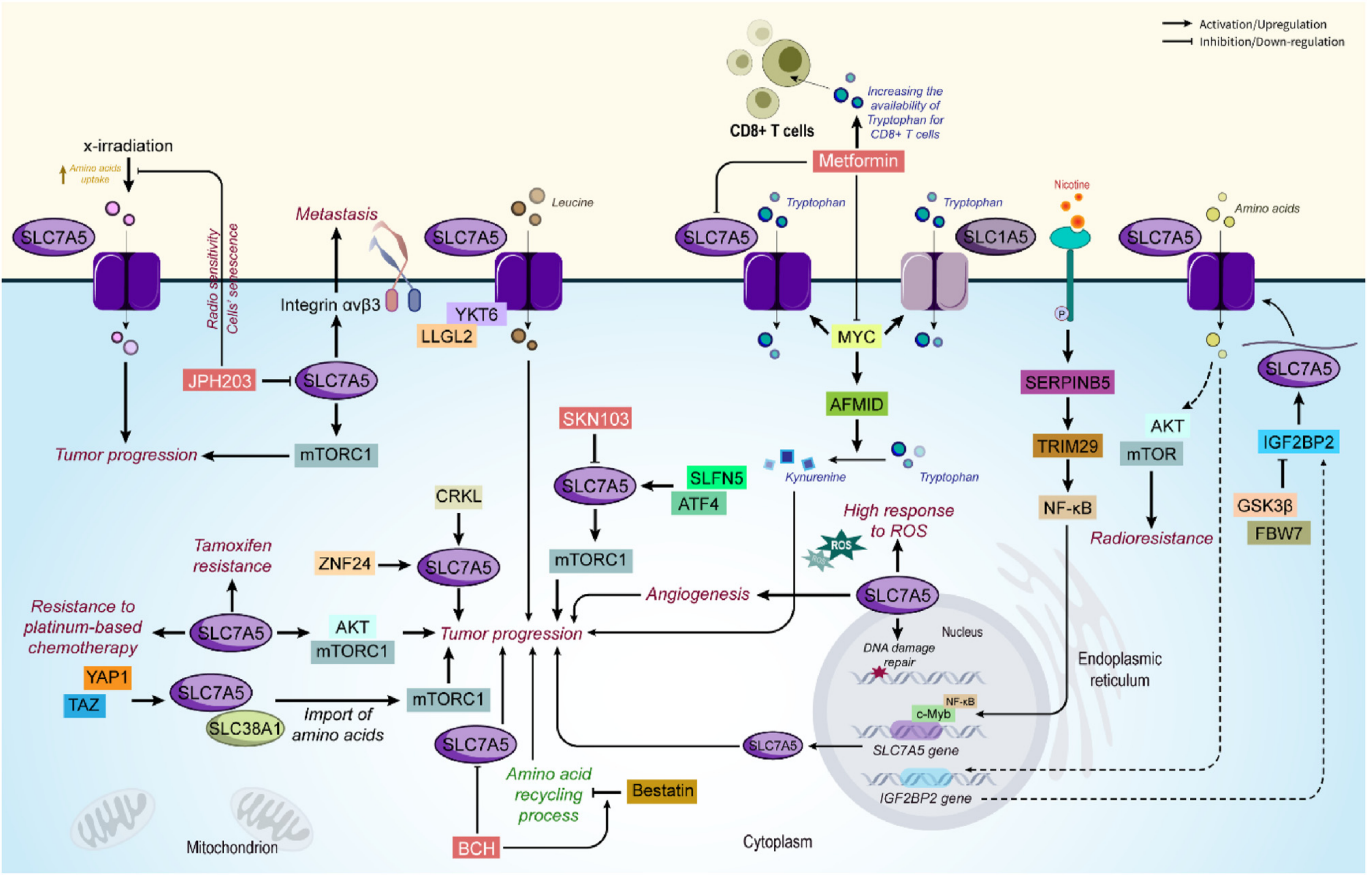
**SLC7A5 role in cancer.** SLC7A5 activity affects mTOR and AKT function in enhancing tumor progression. By regulation of integrins, SLC7A5 also promotes metastasis of cancer cells, a function that can be suppressed by JPH203. In addition, metformin, BCH, and SKN103 are other inhibitors of SLC7A5, while YAP1/TAZ, MYC, and ATF4 augment the expression and function of SLC7A5. Enhancing angiogenesis, DNA damage repair mechanisms, and drug resistance in cancer cells are also mediated by the function of SLC7A5. Abbreviations: AFMID arylformamidase, BCH 2-aminobicyclo[2.2.1]heptane-2-carboxylic acid, ROS reactive oxygen species, SLFN5 Schlafen family member 5, TAZ transcriptional coactivator with PDZ-binding motif, YAP1Yes-associated protein 1.

ZNF24 enhances SLC7A5 expression to promote the growth of KRAS mutant LUAD [124]. LAT-1 expression was also found to be associated with factors involved in the mTOR pathway (including EGFR, a loss of PTEN, p-mTOR, and p-S6K) and hypoxic situation (including HIF-1α, hexokinase I, VEGF, and CD34) in LUAD [125]. Importantly, a study revealed that c-Myc regulates SLC7A5 in pancreatic cancer, and inhibition of c-Myc leads to severe reduction of SLC7A5 protein level. LAT-1 promoter was shown to have a canonical binding sequence for c-Myc and upregulation of c-Myc augments LAT-1 promoter activity [126]. Astatine-211 (^211^At) is a radionuclide for targeted α-therapy. In a study, an anti-cancer therapy with ^211^At-labeled α-methyl-l-tyrosine (^211^At-AAMT) which has a high affinity for LAT-1 was developed as a carrier of ^211^At into tumors. ^211^At-AAMT significantly inhibits tumor cell growth and induces DNA breaks in pancreatic cancer cells [127]. Schlafen family member 5 (SLFN5) is an androgen receptor-regulated protein in castration-resistant prostate cancer (CRPC) that interacts with ATF4 to regulate the LAT-1 transporter. Interestingly, silencing SLFN5 leads to decreased amino acid levels and mTORC1 activity in a LAT1-dependent manner, and subsequently impaired tumor growth [128]. In surgically resected tongue cancer, LAT-1 was one of the amino acid transporters and its high expression level was shown to be associated with disease-staging lymph node metastasis, vascular invasion, and lymphatic permeation. Also, LAT-1 was suggested as an independent prognostic factor of poor prognosis [129]. Also, a high expression level of SLC7A5 was found to be associated with resistance to platinum-based chemotherapy in NSCLC patients with postoperative recurrence [130] (Figure 6).

In an effort, nanosystems were designed to target LAT-1 to assess their uptake by MCF-7 and HeLa cells. In this study, glutamine was conjugated to polyoxyethylene stearate as a ligand to prepare LAT-1-targeting PLGA nanoparticles. Significant cellular uptake and toxicity were demonstrated following the treatment of cancer cells by these nanoparticles and internalized LAT-1 was recycled back to the cell surface within 3 h, providing sufficient transporters for nanoparticle internalization. Also, it was reported that LAT-1 targeting nanoparticles exhibit high accumulation at the tumor site [131]. In another study, LAT1-targeting thermoresponsive fluorescent polymer probes based on poly(N-isopropylacrylamide-co-N,N-dimethylacrylamide) (P(NIPAAm-co-DMAAm)) were synthesized and the result suggested that nanoparticles uptake by HeLa cells was promoted by the LAT1 affinity site [132]. Also, liposomes composed of egg phosphatidylcholine (EPC) and dioleoyl phosphatidylethanolamine modified with LAT1-targeting thermoresponsive polymer, l-tyrosine-conjugated poly(*N*-isopropylacrylamide-*co*-*N*,*N*-dimethylacrylamide) (P(NIPAAm-*co*-DMAAm)) have shown increased uptake by HeLa cells compared with liposomes not modified with l-tyrosine [133]. Furthermore, tyrosine-conjugated ultra-small superparamagnetic iron oxide nanoparticles (USPIONs) were prepared to target LAT-1 in breast cancer. Interestingly, tyrosin-conjugated USPION accumulation at the tumor site was reported to be seven times higher than that of non-targeted USPIONs [134]. Moreover, seed-mediated synthesis of monodispersed multibranched gold nanoparticles (AuNPs) using the catechol-containing LAT-1 ligands, L- and d-dopa, were performed to prepare nano delivery systems targeting breast cancer cells. Importantly, this method is enough that additional conjugation ligands such as peptides or antibodies are not necessary to be applied. Nanoflower-like AuNPs with diameters of 46, 70, and 90 nm were found to have high colloidal stability and biocompatibility, with high intracellular accumulation. Also, for photothermal therapy (PTT), the authors introduced Ag^+^ during the process of synthesis of the nanoparticles to obtain l-dopa functionalized nanourchin-like AuNPs (AuNUs) with strong near-infrared (NIR) absorbance. l-dopa functionalized AuNUs were useful for PTT of the MDA-MB-231 breast cancer cells and increased the sensitivity of cancer cells to cisplatin and docetaxel [135].

### 3.8 SLC7A8

In contrast to most of the SLC transporters which have oncogenic function in cancer, SLC7A8 (LAT-2) was found to have dual functions. For instance, a study reported that SLC7A8 expression is a marker of favorable prognosis in ER+ low proliferative invasive breast cancer. Furthermore, *SLC7A8* mRNA and protein overexpression are associated with good prognostic features, including small tumor size, and low tumor-grade [136]. However, another study showed that SLC7A8 regulates two glutamine-dependent positive feedback loops for activation of the mTOR pathway including LAT2/p-mTOR^Ser2448^ and glutamine/p-mTOR^Ser2448^/glutamine synthetase loop to enhance glycolysis in pancreatic cancer. In addition, SLC7A8 was found to increase gemcitabine resistance in pancreatic cancer [137]. In LUAD, low expression level of SLC7A8 was associated with poor prognosis and immune cell infiltration. However, its overexpression inhibited tumor cell growth and migration [138].

### 3.9 SLC7A11

Among SLCs, a body of studies highlighted the critical function of SLC7A11 in cancer and revealed the underlying mechanisms, upstream and downstream factors, or pathways by which SLC7A11 plays its roles in cancer [[139], [140], [141], [142]].

In NSCLC, SLC7A11 is highly expressed, and its upregulation is associated with advanced stage and worse 5-year survival. Also, inhibition of SLC7A11 with sulfasalazine reduced tumor cell proliferation and invasion [143]. In lung cancer stem-like cells, SRY (sex determining region Y)-box 2 (SOX2) binds to the SLC7A11 promoter to increase its expression, conferring ferroptosis resistance and promoting stemness. This study revealed that SLC7A11, a cystine transporter is overexpressed in lung cancer stem-like cells. Moreover, as it was shown that these cells have a strong capacity for taking cystine, they are more resistant to ferroptosis. Furthermore, the authors have found that SOX2 increases SLC7A11 to protect cancer cells from ferroptosis, and mutations in SOX2 binding sites in *SLC7A11* gene sensitize cancer cells to ferroptosis. In addition, cystine uptake following the upregulation of SLC7A11 is associated with GSH synthesis which also prevents ferroptosis and oxidative modification of SOX2-Cys265 [144]. Regarding its function in importing cystine, SLC7A11 plays a critical role in responding to oxidative stress. Briefly, glutamylcysteine synthetase converts cystine to cysteine, a precursor of GSH, and glutathione synthetase produces GSH which is a co-factor of ROS detoxification enzymes such as glutathione peroxidase 4 (GPX4). In addition, GPX4 uses GSH to suppress ferroptosis through detoxifying lipid hydroperoxides to lipid alcohols [145]. In a study on KRAS-mutant LUAD cells, it was found that SLC7A11 is overexpressed in these cells and mediates tumor progression. Interestingly, an inhibitor of SLC7A11 named HG106 was shown to increase oxidative stress and endoplasmic reticulum-mediated apoptosis. Indeed, this study shows that these types of cells are vulnerable to SLC7A11 inhibition, making SLC7A11 a potential therapeutic target [146]. Kelch-like ECH-associated protein 1 (Keap1), the upstream negative regulator of Nrf2 was found to control ferroptosis in lung cancer cells. Interestingly, KEAP1/Nrf2 controls both SLC7A11-GSH-GPX4 and ubiquinone (CoQ)-ferroptosis suppressor protein 1 (FSP1) arms to defend against ferroptosis. Indeed, by impairment of an arm, cancer cells are still resistant to ferroptosis by using the other arm. Keap1 is an inhibitor of Nrf2 and is mutated or inactivated in lung cancers. Also, FSP1 is the downstream target of Nrf2. Importantly, suppression of CoQ-FSP1 was found to increase the sensitivity of *KEAP1-*deficient lung cancer cells to radiation through inducing ferroptosis [147].

In breast cancer, metformin suppresses the UFMylation process of SLC7A11 to decrease its protein stability and induce ferroptosis in an AMPK-independent manner. In addition, metformin increases the intracellular level of Fe^2+^, and in combination with sulfasalazine, an inhibitor of the x_c_^−^ system, augments ferroptosis and suppresses breast cancer cell proliferation [148]. Although many studies indicated that SLC7A11 overexpression leads to tumor progression, some studies revealed that SLC7A11 downregulation results in resistance to chemotherapy. For instance, a study revealed that silencing SLC7A11 or cystine deprivation leads to increased P-glycoprotein (P-gp), an important factor involved in multiple drug resistance. Indeed, downregulation of SLC7A11 promotes ROS-induced P-gp upregulation and drug resistance in breast cancer cell line [149]. Similarly, another study showed that overexpression of SLC7A11 significantly promotes the sensitivity of ovarian cancer cells to paclitaxel. In addition, SLC7A11 increases the expression of autophagy genes including *LC3*, *Atg16L1*, and *Atg7* on paclitaxel-treated cells in increasing drug sensitivity. However, a low level of SLC7A11 mediates drug resistance via competing endogenous RNA (ceRNA) interactions with autophagy genes [150]. In BRCA mutant ovarian cancer, it was shown that ferroptosis is involved in the efficacy of Poly (ADP-ribose) polymerase (PARP) inhibitor Olaparib drug. Importantly, pharmacological or genetic suppression of PARP reduces SLC7A11 in a p53-dependent manner, accompanied by decreased GSH synthesis and increased lipid peroxidation and ferroptosis [151]. SLC7A11 expression level in CRC stem cells was shown to be higher than that in CRC cells. Erastin, an inhibitor of SLC7A11, was revealed to prevent chemo-resistance through increasing ferroptosis and decreasing stemness [152]. APC Membrane Recruitment Protein 1 (AMER1) is a key regulator of ferroptosis which recruits β-TrCP1/2 to mediate SLC7A11 and ferritin light chain (FTL) ubiquitination and degradation in CRC. Thus, AMER1 induces ferroptosis by binding to SLC7A11 and preventing its activity [153]. In CRC, LPCAT2 mediates acetylation of Protein Arginine Methyltransferase 1 (PRMT1) at the K145 site to suppress its expression and prevent its nuclear translocation. In turn, SLC7A11 which is the PRMT1 downstream target is suppressed and ferroptosis cell death is induced [154].

Interestingly, a long non-coding RNA (lncRNA) named SLC7A11-AS1, an overlapping cis-natural antisense transcript at the *SLC7A11* gene locus, was shown to decrease in gastric cancer. SLC7A11-AS1 directly targets SLC7A11 to decrease its expression and mediate cisplatin-resistance [155]. In addition, SLC7A11 targets PI3K/Akt pathway to inhibit ferroptosis, and promote invasion, metastasis, and proliferation in gastric cancer [156].

A study also revealed the role of SLC7A11 in patients with papillary thyroid carcinoma (PTC). Fat mass and obesity-associated protein (FTO) was shown to be down-regulated in thyroid carcinoma which is responsible for suppressing proliferation, metastasis, and invasion. FTO binds to the putative N6-methyladenosine (m^6^A) sites of SLC7A11 3′UTR to decrease its expression and increase ferroptosis [157]. In PDAC, proper localization of SLC7A11 was shown to rely on autophagy, and impairing autophagy leads to SLC7A11 localization at the lysosome in a mTORC2-dependent manner [158]. Also, overexpression of SLC7A11 in PDAC-derived cancer-associated fibroblasts (CAFs) was demonstrated in a study. SLC7A11 knockdown in CAFs attenuates cell proliferation, increases their senescence, and reduces collagen remodeling and spheroid growth of PDAC *in vitro*. Importantly, genetic ablation of SLC7A11 in PDAC cells was not associated with tumor growth inhibition, but stable inhibition of SLC7A11 was shown to be required to decrease tumor growth and metastasis [159].

Interestingly, SLC7A11 expression in cancer cells is induced by stress-inducible transcription factors, ATF4 and Nrf2, following glucose starvation. SLC7A11 was found to render cancer cells more dependent on glucose and increase the sensitivity of cells to glucose starvation-induced cell death. Indeed, ATF4 and NRF2 bind on the *cis*-regulatory elements of the *SLC7A11* gene to mediate SLC7A11 overexpression and promote glucose starvation-induced cell death. Importantly, it was demonstrated that glutamate efflux regulation by SLC7A11 and intracellular glutamate/αKG levels reduction is responsible for increasing the sensitivity to glucose starvation in SLC7A11-overexpressing cells [160]. SLC7A11 has also a potential role in chemo-resistance by cancer cells. A study has suggested that SLC7A11 expression could be used as a predictor of cellular response to l-alanosine and glutathione-mediated resistance to geldanamycin [161]. Intriguingly, a study showed that moderate and high expression of SLC7A11 has different effects on H_2_O_2_-induced cell death, including protective and sensitizing effects, respectively. In addition, high overexpression of SLC7A11 enhances primary tumor growth but prevents tumor metastasis, likely because SLC7A11-high cancer cells are vulnerable to oxidative stress-induced cell death during metastasis [162]. Furthermore, it was shown that mTORC1 induces endoplasmic reticulum oxidoreductase 1 alpha (ERO1α) activation, which mediates activation of IL-6/signal transducer and activator of transcription 3 (STAT3) pathway. This pathway then increases the transcription of the SLC7A11 gene and results in ferroptosis resistance, tumor cell proliferation, and angiogenesis [163].

**Figure 7:**
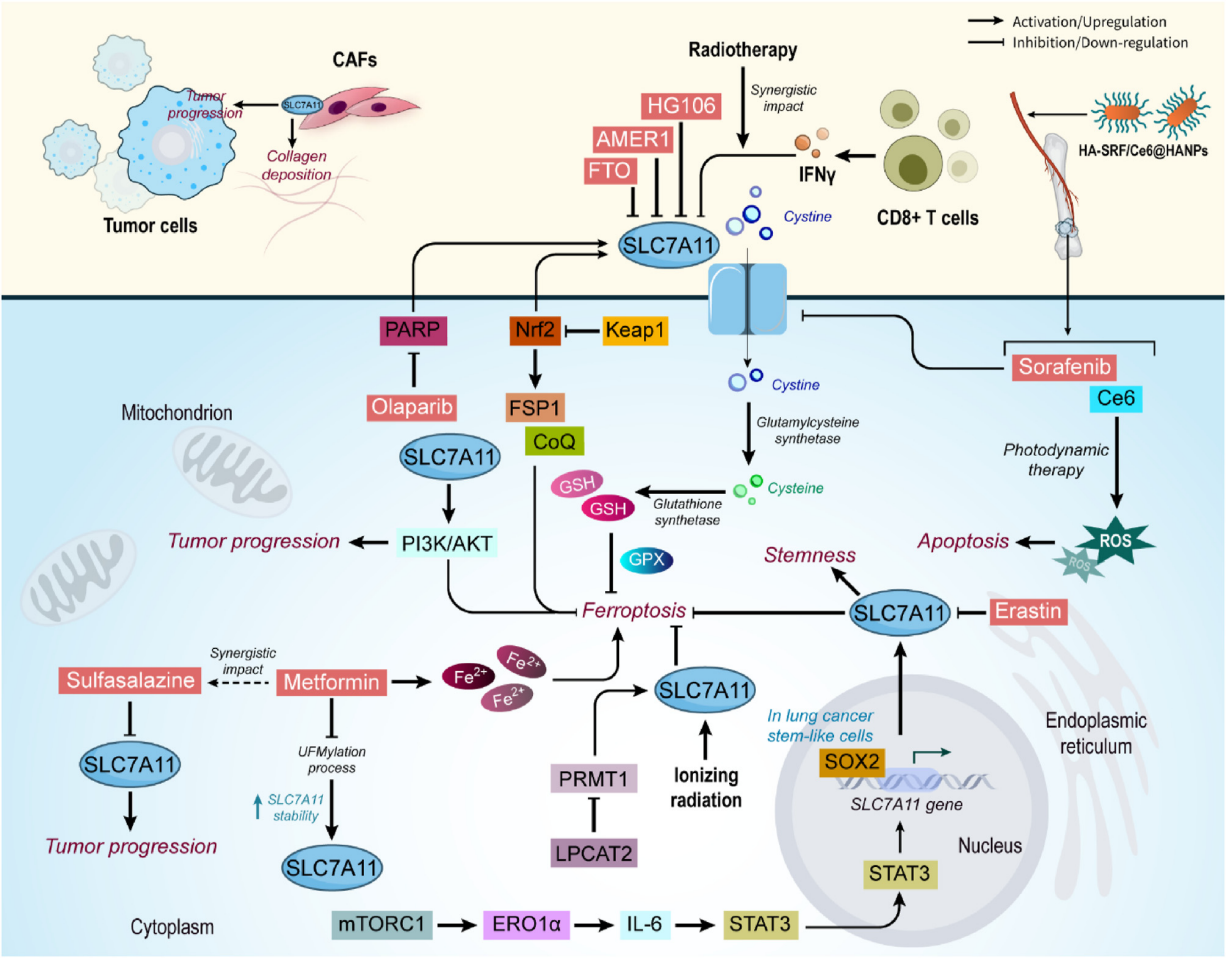
**SLC7A11 role in cancer.** SLC7A11 is an important component of defense against oxidative stress damage. SLC7A11 enhances tumor progression, prevents ferroptosis in cancer cells, and interacts with TME. Metformin, sulfasalazine, and erastin effectively prevent the function of SLC7A11 in cancer, thereby exerting anti-cancer function. Abbreviations: AMER1 APC Membrane Recruitment Protein 1, CAFs cancer-associated fibroblasts, CoQ ubiquinone, FSP1 ferroptosis suppressor protein 1, FTO Fat mass and obesity-associated protein, Keap1 Kelch-like ECH associated protein 1, PARP Poly (ADP-ribose) polymerase, SOX2 SRY (sex determining region Y)-box 2.

Ionizing radiation (IR) is demonstrated to increase the expression of SLC7A11 as a ferroptosis inhibitor and SLC7A11 upregulation subsequently promotes radio-resistance by inhibiting ferroptosis. Interestingly, inhibition of SLC7A11 by ferroptosis inducers sensitizes cancer cells to IR [164]. Similarly, another study revealed the importance of using ferroptosis inhibitors that target SLC7A11 in combination with radiotherapy to treat p53-nutant cancers [165]. It was also demonstrated that immunotherapy has a positive impact on the response of tumor cells to radiotherapy and this effect is induced by enhancing tumor-cell ferroptosis. A study revealed that IFNγ derived from immunotherapy-activated CD8^+^ T cells act synergistically with radiotherapy to inhibit SLC7A11 which results in reduced cystine uptake, and increased tumor lipid oxidation and ferroptosis, thereby tumor suppression [166]. In an effort, mesoporous hydroxyapatite nanoparticles functionalized with hyaluronic acid were used to deliver sorafenib and photosensitizer chlorin e6 (HA-SRF/Ce6@HANPs). This delivery system was found to effectively suppress breast cancer bone metastasis via the synergistic function of sorafenib which suppresses SLC7A11, and photodynamic therapy induced by 660 nm laser which increases ROS level. Together, HA-SRF/Ce6@HANPs finally induce apoptosis and decrease cancer-induced bone pain [167] (Figure 7) (Table 1).

**Table 1:** The role of SLC7A11 in cancer through regulating ferroptosis.

Cancer type	Upstream regulator	Important remarks	Ref.
Lung cancer	RBMS1	RBMS1 is elevated in lung cancer. RBMS1 interacted with eIF3d to promote *SLC7A11* translation.	[168]
	Dihydroartemisinin	Dihydroartemisinin inhibits ferroptosis by inactivating PRIM2/SLC7A11 axis.	[169]
	EGCG	EGCG restrained tumor progression induced by leptin through targeting STAT1/SLC7A11.	[170]
	Butyrate	Butyrate promotes erastin-induced ferroptosis by regulating the ATF3/SLC7A11 pathway.	[171]
NSCLC	Sinapine	Sinapine induces ferroptosis through p53-dependent downregulation of SLC7A11.	[172]
	AhR	AhR is highly expressed in NSCLC tissue. AhR binds to the promoter region of SLC7A11 promotes, increases its expression, and inhibits ferroptosis in increasing tumor progression.	[173]
	Capsaicin	Capsaicin induces ferroptosis by inactivating SLC7A11/GPX4 signaling.	[174]
LUAD	METTL3	METTL3 recruits YTHDF1 and directly targets SLC7A11 to mediate its mRNA stabilization through m6A modification. SLC7A11 targeting by METTL3 leads to tumor cell proliferation and ferroptosis inhibition.	[175]
Esophageal squamous cell carcinoma	NRF2	NRF2 binds to the promoter region of SLC7A11 to induce its expression, promote radio-resistance, and reduce radiotherapy-related ferroptosis morphologic features.	[176]
Osteosarcoma	KDM4A	KDM4A is upregulated in osteosarcoma, and its expression is associated with poor prognosis, tumor progression, and lung metastasis. KDM4A mediates H3K9me3 demethylation of SLC7A11 to regulate SLC7A11 transcription and ferroptosis.	[177]
CRC	Benzopyran derivative 2-imino-6-methoxy-2H-chromene-3-carbothioamide (IMCA)	IMCA regulated the activity of the AMPK/mTOR/p70S6k signaling pathway and decreased the expression of SLC7A11 to increase ferroptosis.	[178]
	AADAC	AADAC inhibits lipid peroxidation in a SLC7A11-dependent manner to prevent CRC cell liver metastasis from ferroptosis.	[179]
	PERK	PERK increases the expression of SLC7A11 to block ferroptosis.	[180]
	ALKBH5	ALKBH5 is downregulated in CRC and its upregulation mitigates tumor growth. ALKBH5 erased the m6A modification on *SLC7A11* mRNA to reduce its mRNA stability and expression, and to promote ferroptosis.	[181]
	Sodium butyrate	Sodium butyrate induces ferroptosis through CD44/SLC7A11 axis.	[182]
	FTO	FTO increases the expression of SLC7A11 and GPX4 in an m^6^A-YTHDF2-dependent manner to prevent ferroptosis.	[183]
CRC stem cells	Vitamin D	Vitamin D induces ferroptosis by downregulating SLC7A11.	[184]
Colon cancer	Pt3R5G	Pt3R5G decreased the expression of SLC7A11 to increase ferroptosis.	[185]
	HECTD3	HECTD3 endogenously interacts with SLC7A11 protein to mediate its polyubiquitination and increase the degradation of SLC7A11 proteins, thereby increasing ferroptosis.	[186]
	Plumbagin	Plumbagin increases the expression and phosphorylation of p53 to decrease the mRNA and protein levels of SLC7A11. Plumbagin increases Fe^2+^ level and reduces GSH and GPX4.	[187]
Gastric cancer	Tanshinone IIA	Tanshinone IIA induces ferroptosis through p53-mediated SLC7A11 downregulation.	[188]
	TRIM7	TRIM7 interacts with SLC7A11 through its B30.2/SPRY domain and promotes Lys48-linked polyubiquitination of SLC7A11, which inhibits SLC7A11/GPX4 axis and induces ferroptotic cell death.	[189]
Breast cancer	ESR1	ESR1 increases the expression of SLC7A11 at the early stage after IR. ESR1/SLC7A11 knockdown significantly promotes radiation-induced ferroptosis in ER-positive breast cancer cells.	[190]
	Sculponeatin	Sculponeatin is a diterpenoid extracted from Isodon sculponeatus. Sculponeatin reduced the expression of ETS1 which is an inducer of SLC7A11-dependent ferroptosis in breast cancer. Sculponeatin binds to the ETS domain of ETS1 to inhibit the transcription of SLC7A11. Sculponeatin mediates proteasomal degradation of ETS1 by inducing SYVN1-mediated ubiquitination.	[191]
	Fascin	Fascin directly interacts with SLC7A11 to reduce its stability via ubiquitin-mediated proteasome degradation and enhances the vulnerability of cancer cells to erastin-induced ferroptosis.	[192]
Ovarian cancer	CEBPG	CEBPG is upregulated in ovarian cancer. CEBPG regulates the transcription of *SLC7A11* to suppress ferroptosis.	[193]
	HRD1	HRD1 is downregulated in ovarian cancer. Overexpression of HRD1 is associated with tumor growth and proliferation suppression, and increased apoptosis and ferroptosis. For exerting its functions, HRD1 interacts with SLC7A11 and increases its degradation.	[194]
Endometrial cancer	Sodium butyrate	Sodium butyrate promotes the expression of RBM3 to decrease SLC7A11 expression, thereby enhancing ferroptosis.	[195]
HCC	C8orf76	C8orf76 binds to the promoter of the *SLC7A11* gene to increase its expression and suppress ferroptosis. C8orf76 upregulation increased the resistance to lipid disturbance and ferroptosis induced by erastin or sorafenib.	[196]
	Aspirin	Aspirin induces ferroptosis by inhibiting NF-κB p65-activated SLC7A11 transcription.	[197]
	ABCC5	ABCC5 expression is high in sorafenib-resistant HCC cells. ABCC5 stabilizes SLC7A11 to increase GSH levels, decrease lipid peroxidation, and inhibit ferroptosis.	[198]
Gallbladder cancer	RUNX3	RUNX3 aberrantly downregulated in gallbladder cancer. Downregulation of RUNX3 results from DNA methylation caused by DNA Methyltransferase 1. RUNX3 induces ferroptosis through activating ING1 transcription, thereby repressing SLC7A11 in a p53-dependent manner.	[199]
Cholangiocarcinoma	SHARPIN	SHARPIN, a component of the linear ubiquitin chain activation complex is increased in cholangiocarcinoma. Silencing of SHARPIN suppresses the ubiquitination and degradation of p53 and decreases the levels of SLC7A11, GPX4, SOD-1, and SOD-2, which leads to excessive oxidative stress and ferroptosis.	[200]
Bladder cancer	PHGDH	PHGDH is highly expressed in patients with bladder cancer. PCBP2 stabilizes *SLC7A1*1 mRNA and increases its expression to suppress ferroptosis. NCT-502, a PHGDH inhibitor, increases ferroptosis and suppresses bladder cancer progression.	[201]
	P53	P53 inhibits SLC7A11 to activate lipoxygenase activity of ALOX15B to trigger ferroptosis.	[202]
ccRCC	MITD1	MITD1 is overexpressed in ccRCC. Silencing MITD1-induced ferroptosis and suppressed tumor growth and migration through the TAZ/SLC7A11 pathway.	[203]
Prostate cancer	Antimony	Antimony is an environmental pollutant. A low dose of antimony increases the expression of Nrf2, SLC7A11, and GPX4, suppresses ferroptosis, and promotes prostate cancer progression.	[204]
PTC	ETV4	ETV4 expression is increased in PTC tissues and cells. Silencing ETV4 blocked PTC cell proliferation and cell cycle progression. ETV4 increased *SLC7A11* transcription by binding to its promoter region directly to suppress ferroptosis.	[205]
–	OTUB1	OTUB1 is overexpressed in human cancers. OTUB1 interacts with and stabilizes SLC7A11. CD44 promotes the interaction between SLC7A11 and OTUB1 to enhance the stability of SLC7A11. CD44 suppresses ferroptosis in an OTUB1-dependent manner.	[206]
	Sorafenib combined with ursolic acid	Sorafenib/ursolic acid induces Mcl-1-related apoptosis and SLC7A11-dependent ferroptosis.	[207]
	BAP1	BAP1 inhibits SLC7A11 through reducing H2A ubiquitination	[208]

### 3.10 SLC17A7

Interestingly, it was found that although SLC17A7 is downregulated in glioblastoma (GBM) compared with normal brain tissues, it suppresses GBM cell proliferation, invasion and metastasis [209]. In osteosarcoma, the expression level of SLC17A7 was shown to increase in response to romidepsin, a histone deacetylase (HDAC) inhibitor. The authors reported that HDAC inhibition decreased glutamate secretion from osteosarcoma cells in impairing tumor viability [210].

### 3.11 SLC43A1

The importance of SLC43A1 in cancer is well recognized in a study by Yue et al. the authors showed that MYC binds to the specific E box elements within genes of *SLC43A1* and *SLC7A5* to increase their expression and localization on the cell surface. Then, essential amino acids are imported by SLC43A1 and SLC7A5, and *Myc* mRNA translation is amplified. Also, glucose and glutamine metabolism are dependent on SLC43A1 and SLC7A5. Importantly, essential amino acids attenuate general control nonrepressed-2 (GCN2)-eukaryotic initiation factor 2a (eIF2a)-ATF4 amino acid stress response pathway to increase *Myc* mRNA translation. By this feedforward regulatory loop, oncogenic MYC enhances amino acid metabolism and tumorigenesis [211]. SLC43A1 or LAT-3 was shown to be overexpressed in androgen receptor-expressing prostate cancer. It was also found that dihydrotestosterone and bicalutamide increase and decrease LAT-3 expression, respectively. Silencing LAT-3 leads to inhibition of cell proliferation, invasion, and metastasis. In addition, androgen receptor and LAT-3 knocking down were associated with reduced LAT-3 and mTOR expression, respectively. In addition to mTOR, Separase (ESPL1) is another target of LAT-3 which is involved in the cancer cell cycle, and suppression of LAT-3 leads to cell cycle arrest and decreases the number of cells in the S and G2/M phases [212]. Also, another study revealed that EGF-activated PI3K/Akt/mTORC1 signaling controls leucine import through LAT-3 in prostate cancer cells, providing a condition by which the cells rapidly uptake amino acids to promote cell growth [213]. Interestingly, a novel monoterpene glycoside named ESK246, derived from the plant *Pittosporum venulosum*, suppresses prostate cancer cell proliferation by inhibiting LAT-3 and subsequently mTORC1 signaling [214].

### 3.12 SLC43A2

**Figure 8:**
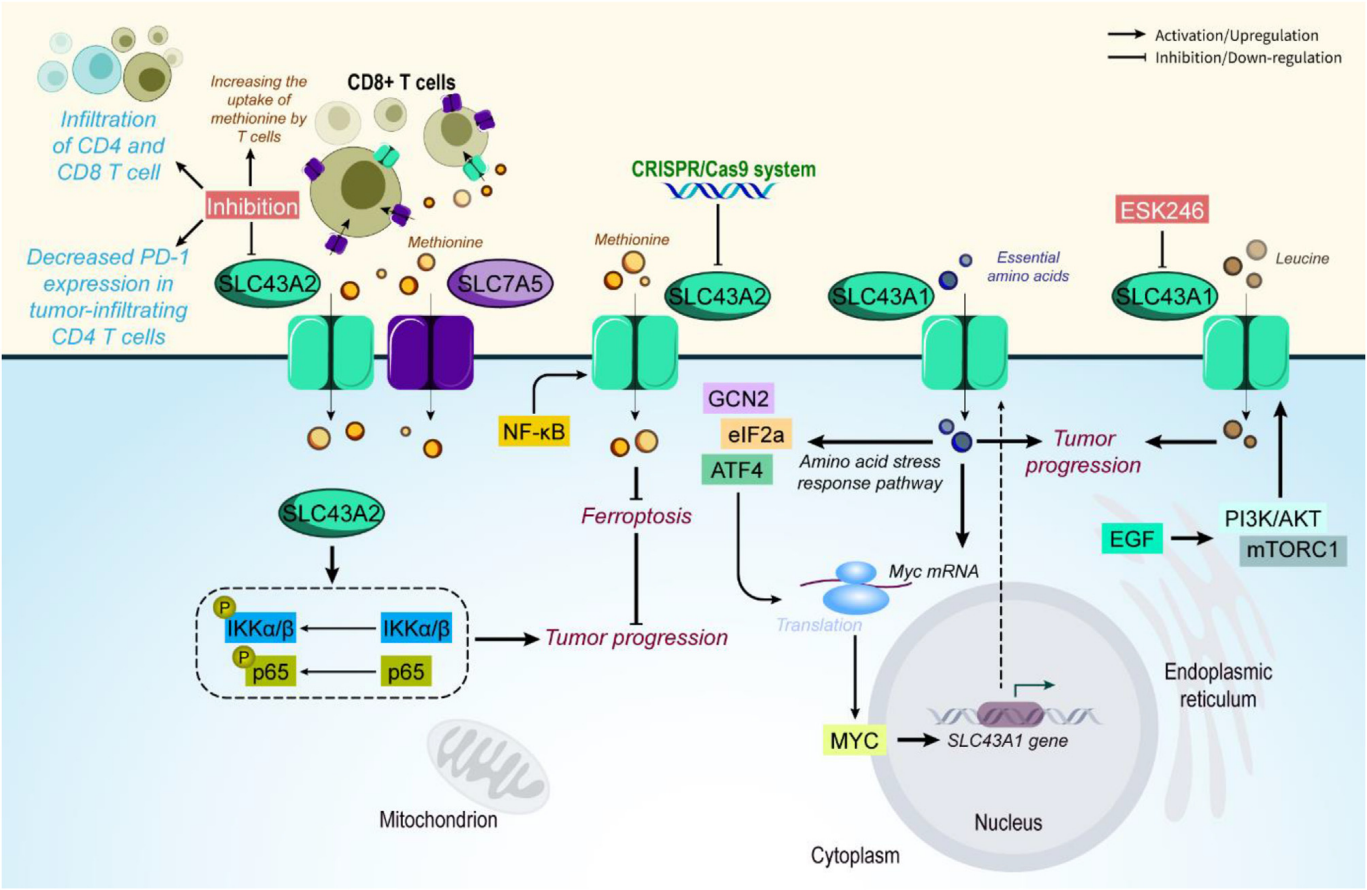
**SLC43A1 and SLC43A2 role in cancer.** SLC43A1 and SLC43A2 exert oncogenic function in cancer cells and TME. They interact with various molecules and pathways and their function is affected by therapeutic and oncogenic molecules. Abbreviations: eIF2a eukaryotic initiation factor 2a, ESPL1 Separase, GCN2 general control nonrepressed-2, mTORC1 mammalian target of rapamycin complex 1.

SLC43A2, a methionine transporter, was found to exert an oncogenic role in esophageal squamous cell carcinoma. Importantly, SLC43A2 increases the phosphorylation of IKKα/β and p65, and NF-κB silencing leads to inhibition of SLC43A2 and GPX4 in both mRNA and protein levels, thereby downregulation of methionine uptake. Following these changes, ferroptosis and apoptosis are induced and esophageal squamous cell carcinoma progression is impaired [215]. In a novel study, CRISPR/Cas9 system carrying in nanoplatforms was used to decrease methionine intake by cancer cells via inhibiting SLC43A2. For constructing CRISPR/Cas9 plasmid (pDNA), sgRNA targeting *SLC43A2* was cloned into plasmid PX330. Mixing pDNA during the process of mixing metal ions and organic ligands makes the pDNA more firmly, because pDNA is encapsulated in the whole nanoparticles instead of the unstable surface adsorption. SLC43A2 inhibition relieved the methionine competition pressure of T cells; concurrently, the released nutrition metal ions such as Zn and Mn ions activate the cGAS/STING pathway. Promoting the STING pathway mediates immunity and facilitates the development of amino acid metabolic intervention-based cancer therapy. Indeed, STING upregulation leads to increased TBK1, and then IFN-β upregulation, which mediates dendritic cell maturation and T cell activation. These changes accompanied by increased access to methionine help T cells to inhibit tumor progression andenhanceg tumor elimination [216]. Intriguingly, it was found that SLC43A2 and SLC7A5 are expressed by tumor cells, but T cells mostly expressed SLC7A5. Silencing SLC43A2 in a mouse melanoma cell line was shown to enable T cells to uptake more methionine in competition with tumor cells. As an inhibitor that specifically targets SLC43A2 is not available, suppressing all the L-type amino acid transporters inhibits methionine uptake by both T cells and cancer cells and also inhibits CD8 T ccellinfiltration [217]. Similarly, a study showed that SLC43A2 deficient cells are associated with delayed growth after tumor transplantation in mice, while no differences were seen in the *in vitro* proliferation. In addition, the infiltration of CD4 and CD8 T cells and release of IFN-γ by these cells was found to increase in SLC43A2 deficient tumors. Moreover, PD-1 expression in tumor-infiltrating CD4 T cells from SLC43A2 deficient tumors was reported to be decreased [218] (Figure 8).

### 3.13 SLC43A3

SLC43A3 a novel fusion gene NUP160–SLC43A3 was revealed to be overexpressed in angiosarcoma specimens. In tumors harboring the fusion gene, more rapid tumor progression and shortened duration between symptoms' unset and the first hospital visit were reported [219]. Also, another study found that *SLC43A3*-encoded equilibration nucleobase transporter 1 (ENBT1) is responsible for importing 6-mercaptopurine, an agent used for the treatment of acute lymphoblastic leukemia, into leukemia cells [220]. In addition to the SLCs, some transporters are not studied well in cancer (Table 2), and future studies could reveal their critical function in tumorigenesis.

**Table 2:** SLC proteins with their role in cancer.

SLC family	Subgroup	cDNA	Substrates	Cancer type	Function in cancer	Ref.
SLC1						
	SLC1A1	EAAC1, EAAT3	Glutamate, aspartate, cysteine	Lung cancer	Oxidative stress-mediated cell death	[221]
	SLC1A2	EAAT2	Glutamate, aspartate	Gastric cancer	Tumor growth and survival	[222]
	SLC1A3		Glutamate, aspartate	CD133+ thyroid cancer cells	Self-renewal capacity	[223]
	SLC1A4	ASCT1	Alanine, serine, cysteine, threonine	Prostate cancer	Tumor growth	[224,225]
	SLC1A5	ASCT2	Alanine, serine, cysteine, threonine, glutamine, asparagine	CRC	Tumor growth, survival, proliferation, and apoptosis inhibition	[226]
				Gastric cancer	Local invasion, higher lymph node metastasis, and proliferation	[227]
				Esophageal cancer	Cell cycle progression and apoptosis inhibition	[228]
				NSCLC	Shorter overall survival, and lymph node metastasis	[229]
	SLC1A6	EAAT4	Glutamate, aspartate	Nasopharyngeal carcinoma	Cisplatin resistance	[230]
SLC3						
	SLC3A1	rBAT	Cysteine	Breast cancer	Tumor growth	[231]
	SLC3A2	CD98hc	Leucine, methionine, isoleucine, valine, Phenylalanine, threonine, tryptophan	Gastric cancer	Biomarker for molecular imaging-based detection of gastric cancer	[232]
				HNSCC	Radio-resistance	[233]
				CRPC	EMT, cell cycle progression and apoptosis inhibition	[234]
				Triple negative breast cancer (TNBC)	Tumor spheroid growth	[235]
SLC7						
	SLC7A1	CAT-1	Arginine, lysine, ornithine	CRC	Tumor growth	[236]
	SLC7A5	LAT-1	Leucine, methionine, isoleucine, valine, phenylalanine, tyrosine, tryptophan	Highly proliferative ER+ breast cancer	Poor patient outcome	[237]
				CRC	Tumor cell proliferation	[238]
					Tumor cell proliferation, poor prognosis, and oxaliplatin resistance	[239]
				Invasive breast cancer	Independent poor prognostic factor	[240]
				Hormone resistant prostate cancer	Tumor cell proliferation, invasion, and migration	[241]
				Oral squamous cell carcinoma	Tumor growth	[242]
				Ovarian carcinoma	Poor prognosis, and chemoresistance	[243]
				Thymic carcinoma	Tumor growth	[244]
				Gastric carcinomas	Poor prognosis, and tumor cell proliferation	[245]
				Pulmonary pleomorphic carcinoma	Independent prognostic factors for predicting worse outcome, tumor proliferation, and advanced stage	[246]
				Liver cancer	Tumor proliferation	[247]
				Esophageal squamous cell carcinoma	Lymphatic permeation, and invasion	[248]
	SLC7A9	BAT1	Cysteine	ESCC	Biomarker for lymph node metastasis	[249]
SLC17						
	SLC17A1		Glutamate	Acute myeloblastic leukemia	Resistance to cytosine arabinoside-mediated apoptosis	[250]
	SLC17A2		Glutamate	HCC	Immune infiltration, and prognostic factor	[251]
	SLC17A9		Glutamate	CRC	Prognostic biomarker	[252]
				ccRCC	EMT, tumor growth, and resistance to vorinostat	[253]

## 4 SLCs in clinical trials

More than 30 SLC proteins have been offered as potential drug targets [254]. However, in a systematic analysis, César-Razquin et al. have suggested that SLCs are the most neglected group of genes in the human genome, despite their clear relevance to human health and disease [255].

It bears noting that although some inhibitors of SLC proteins are recognized, they have not been studied yet through clinical trials. For instance, dl-threo-beta-benzyloxyaspartate (dl-TBOA) is a potent inhibitor of SLC1A3 [256], but its safety and effectiveness are not evaluated in clinical trials. Similarly, studies have revealed that phenylglycine inhibits SLC1A4 (ASCT1) and SLC1A5 (ASCT2) [257]. Likewise, L-4-fluorophenylglycine was shown to significantly inhibit ASCT1 and ASCT2 in rodent models of schizophrenia and visual dysfunction [258]. In addition, there is no selective inhibitor for some of the SLCs, such as SLC7A1, and their development in the future seems promising [87].

However, its clinical applications need to be addressed. In phase I clinical trials, patients with advanced or metastatic cancers with high LAT1 expression were treated with QBS10072S. QBS10072S is a novel non-cleavable compound that inhibits brain metastasis and leptomeningeal metastasis. QBS10072S is recognized and transported by LAT1 across the blood–brain barrier and specifically targets cancer cells [259]. Also, in glioblastoma xenograft models, it was found that QBS10072S has the potential to treat temozolomide-resistant and recurrent glioblastoma [260]. Another study revealed that QBS10072S and QBS10096S reduce the survival of T-cell lymphoma lines *in vitro* and *in vivo* [261]. As the only LAT-1 inhibitor being studied in clinical trials, JPH203 was shown to effectively prevent biliary tract cancer in the first-in-human phase I study of JPH203 (UMIN000016546) [262]. Table 3 provides some of the completed, current, and prospective clinical trials on SLC inhibitors.

**Table 3:** Clinical trials on SLCs registered in ClinicalTrials.gov.

SLC name	Protein name	Substrates	Inhibitor name	Disease	Phase	ID
SLC3A2	4F2hc	Amino acid	IGN523	AML	I	NCT02040506
SLC5A1	SGLT1	Glucose	Sotagliflozin	Type 2 diabetes mellitus	III	NCT02926950
SLC6A9	GlyT1	Neurotransmitter	RO4917838 (Bitopertin)	OCD	II	NCT01674361
			Pf-04958242	Cognitive impairment associated with schizophrenia	II	NCT02855411
SLC7A11	xCT	Amino acid	Sulfasalazine	CRC	III	NCT06134388
				Glioblastoma	I	NCT04205357
				AML	I	NCT05580861
SLC9A3	NHE3	Na/H exchanger	Tenapanor	Irritable bowel syndrome	III	NCT02727751
SLC16A1	MCT1	Amino acid	AZD3965	Lymphoma	I	NCT01791595
SLC18A2	VMAT2	Neurotransmitter	NBI-98854 (Valbenazine)	Tardive dyskinesia	III	NCT02274558
				Tourette syndrome	II	NCT02879578
			Lobeline sulfate	ADHD	II	NCT00664703

## 5 Conclusion and future perspective

This review elucidated the intricate mechanisms by which SLC transporters influence cancer development and progression. By modulating nutrient availability, SLCs play a dual role in both supporting cancer cell growth and rendering tumors vulnerable to therapeutic interventions. We explore the current landscape, highlighting the potential of targeting SLC transporters as a promising avenue for innovative cancer therapies.

SLC transporters constitute a diverse and extensive superfamily of membrane proteins that are pivotal for maintaining cellular homeostasis through the regulation of nutrient transport across biological membranes. In the cancer milieu, SLC proteins have emerged as central players in the metabolism. One of the hallmark features of cancer cells is their increased demand for nutrients to sustain their rapid proliferation and growth. SLC transporters, especially those responsible for the uptake of amino acids, are frequently upregulated in cancer cells to meet these heightened metabolic needs. Amino acid transporters such as SLC1A5 and SLC7A11 are instrumental in supplying cancer cells with the necessary building blocks for protein synthesis, energy production, and other biosynthetic pathways. SLC1A5 overexpression enhances the uptake of amino acids, particularly glutamine, which is a critical substrate for cancer cell growth and proliferation. Also, SLC1A5 mediates metabolic rewiring in cancer cells by importing glutamine, fueling the TCA cycle, and generating ATP to meet their increased energy demands.

In addition, cystine import by SLC7A11 facilitates the maintenance of redox balance. Thereby, these metabolic alterations support the function of cellular signaling pathways that promote cancer cell survival and resistance to therapeutic interventions. In addition, the SLC43A2 transporter is widely expressed in different tissues and is noteworthy for its role in modulating the availability of essential amino acids within the TME, a complex milieu consisting of cancer cells. Cancer cells often exhibit upregulated SLC43A2 expression to fuel their nutrient acquisition, mediate tumor–stromal interactions, and have therapeutic implications. Moreover, it is demonstrated that SLC43A2 expression in cancer cells is more than that in T cells, making SLC43A2 an intriguing target for further investigation in the context of cancer therapy. Importantly, upregulation of SLC7A5 and SLC43A1/2 occurs in certain cancer types, where they increase the uptake of large neutral amino acids such as leucine. Not only critical for protein synthesis, leucine also activates the mTOR pathway, a key regulator of cancer cell growth and metabolism.

SLC3A2 forms a heterodimeric complex with specific SLC7 family such as SLC7A5 and SLC7A11, enhancing the uptake of amino acids. Also, SLC3A2 plays a role in T cell proliferation and activation, which are critical components of immune response. Thus, strategies aimed at inhibiting SLC3A2 or the associated amino acid transporters may disrupt the nutrient supply to cancer cells, making them more susceptible to treatment. SLC7A1 primarily transports cationic amino acids, such as arginine, essential for protein synthesis, cellular growth, and importantly, the production of molecules like nitric oxide which has diverse roles in the body, including vascular function and immune responses.

The dysregulation of SLCs is observed across various cancer types and is often associated with poor prognosis and aggressive disease behavior. Therefore, targeting these transporters has garnered significant interest as a potential therapeutic strategy in oncology. Recent advancements in the development of small molecule inhibitors and targeted therapies against specific SLCs hold promise for disrupting cancer cell nutrient uptake and sensitizing tumors to conventional treatments.

In conclusion, SLCs constitute a critical component of cancer biology by governing nutrient transport, metabolic reprogramming, and therapy resistance. Further research into the precise mechanisms of SLC transporters' regulation and their roles in different cancer contexts is essential for developing new therapeutic approaches with the potential to improve cancer patient outcomes.

## Availability of data and materials

Not applicable.

## Funding

A.P.K. is supported by grants from the 10.13039/501100001459Singapore Ministry of Education (MOE-T2EP30120-0016) and from the NUHS Seed Fund (NUHSRO/2023/039/RO5+6/Seed-Mar/04).

## CRediT authorship contribution statement

**Kiavash Hushmandi:** Writing – original draft, Visualization, Validation, Software, Methodology, Investigation, Formal analysis, Data curation, Conceptualization. **Behzad Einollahi:** Writing – original draft, Validation, Methodology, Investigation, Formal analysis, Data curation. **Seyed Hassan Saadat:** Writing – original draft, Visualization, Methodology, Investigation, Data curation. **E Hui Clarissa Lee:** Writing – original draft, Visualization, Methodology, Investigation, Formal analysis, Data curation. **Marzieh Ramezani Farani:** Writing – review & editing, Writing – original draft, Visualization, Validation, Methodology, Investigation. **Elena Okina:** Writing – review & editing, Visualization, Validation. **Yun Suk Huh:** Writing – review & editing, Visualization, Validation, Software. **Noushin Nabavi:** Writing – review & editing, Visualization, Validation. **Shokooh Salimimoghadam:** Writing – review & editing, Visualization, Validation, Formal analysis, Data curation. **Alan Prem Kumar:** Writing – review & editing, Supervision, Project administration, Investigation, Funding acquisition, Formal analysis, Conceptualization.

## Declaration of competing interest

Authors have no competing interest.

## Data Availability

No data was used for the research described in the article.

## Abbreviations

^211^At: Astatine-211
^211^At-AAMT: ^211^At-labeled α-methyl-l-tyrosine
4F2hc: 4F2 cell surface antigen heavy chain
AADAC: arylacetamide deacetylase
ADHD: attention deficit hyperactivity disorder
AFMID: arylformamidase
AhR: aryl hydrocarbon receptor
ALDH2: aldehyde dehydrogenase 2
ALYREF: Aly/REF export factor
AMER1: APC Membrane Recruitment Protein 1
ASCT2: alanine, serine, cysteine-preferring transporter 2
ASS1: argininosuccinate synthase 1
AuNPs: gold nanoparticles
C8orf76: chromosome 8 open reading frame 76
CAFs: cancer-associated fibroblasts
CAR: chimeric antigen receptor
CAT-1: cationic amino acid transporter-1
ccRCC: clear cell renal carcinoma cells
ceRNA: COMPETING endogenous RNA
CoQ: ubiquinone
CRC: colorectal cancer
CRPC: castration-resistant prostate cancer
DDR1: discoid protein domain receptor 1
EGCG: epigallocatechin gallate
EGFR: epidermal growth factor receptor
eIF2a: eukaryotic initiation factor 2a
ENBT1: equilibrative nucleobase transporter 1
EPC: egg phosphatidylcholine
ER: estrogen-receptor
ERO1α: endoplasmic reticulum oxidoreductase 1 alpha
ESPL1: separase
ETC: electron transport chain
ETV4: E26 transformation specific (ETS) variant 4
FKBP1A: FK506-binding protein 1A
FSP1: ferroptosis suppressor protein 1
FTL: ferritin light chain
FTO: fat mass and obesity-associated protein
GCN2: amino acids attenuate general control nonrepressed-2
GPX4: glutathione peroxidase 4
GSH: glutathione
HCC: hepatocellular carcinoma
HDAC: histone deacetylase
HECTD3: homologous to the E6-associated protein carboxyl terminus domain containing 3
HIF: hypoxia-inducible factor
HNSCC: head and neck squamous cell carcinoma
HOXA13: Homeo box A13
HRD1: 3-hydroxy-3-methylglutaryl reductase degradation
IFNγ: interferon gamma
IMA: invasive mucinous adenocarcinoma
IMCA: 2-imino-6-methoxy-2H-chromene-3-carbothioamide
ING1: inhibitor of growth 1
IR: ionizing radiation
Keap1: Kelch-like ECH associated protein 1
LAT1: L-type amino acid transporter 1
LncRNA: long non-coding RNA
LUAD: lung adenocarcinoma
LUSC: lung squamous cell carcinoma
m6A: N6-methyladenosine
METTL3: methyltransferase-like 3
MIF: migration inhibitory factor
MITD1: MIT-domain containing protein 1
MMP: metalloproteinase
mTORC1: mammalian target of rapamycin complex 1
NFE2L3: nuclear factor erythroid 2-related factor 3
NIR: near-infrared
NRG1: neuregulin
NSCLC: non-small cell lung cancer cell
OCD: obsessive compulsive disorder
PARP: poly (ADP-ribose) polymerase
PDAC: pancreatic ductal adenocarcinoma
P-gp: P-glycoprotein
PHGDH: phosphoglycerol dehydrogenase
PRL: prolactin
PRMT1: protein Arginine Methyltransferase 1
Pt3R5G: petunidin 3-O-[rhamnopyranosyl-(trans-p-coumaroyl)]-5-O-(β-d-glucopyranoside)
PTT: photothermal therapy
RCC: renal cell carcinoma
RIOK3: Rio Kinase 3
ROCK: Rho kinase
ROS: reactive oxygen species
RUNX3: runt-related transcription factor 3
SCRIB: scribble
SHARPIN: shank-associated RH domain interacting protein
SLC: solute carrier
SLFN5: schlafen family member 5
SOX2: SRY (sex determining region Y)-box 2
SPOP: speckle-type BTB/POZ
STAT3: signal transducer and activator of transcription 3
SYVN1: synoviolin 1
TALDO1: transaldolase 1
TAMs: tumor-associated macrophages
TAZ: PDZ-binding motif
TCA: tricarboxylic acid
TME: tumor microenvironment
TNBC: triple-negative breast cancer
TRIM6: tripartite motif 6
TRIM7: tripartite motif-containing protein 7
USPIONs: ultra-small superparamagnetic iron oxide nanoparticles
VMAT2: vesicular monoamine transporter 2
YAP1: yes-associated protein 1
YTHDC2: YT521-B homology containing 2
YTHDF1: YT521-B homology (YTH) domain family of proteins
ZEB1: zinc finger E-box-binding homeobox 1
